# MMP12 serves as an immune cell–related marker of disease status and prognosis in lung squamous cell carcinoma

**DOI:** 10.7717/peerj.15598

**Published:** 2023-08-16

**Authors:** Wei Zhang, Guo-Sheng Li, Xiang-Yu Gan, Zhi-Guang Huang, Rong-Quan He, Hong Huang, Dong-Ming Li, Yu-Lu Tang, Deng Tang, Wen Zou, Jun Liu, Yi-Wu Dang, Gang Chen, Hua-Fu Zhou, Jin-Liang Kong, Hui-ping Lu

**Affiliations:** 1 Department of Pathology, The First Affiliated Hospital of Guangxi Medical University, Nanning, Guangxi Zhuang Autonomous Region, China; 2 Department of Cardiothoracic Surgery, The First Affiliated Hospital of Guangxi Medical University, Nanning, Guangxi Zhuang Autonomous Region, China; 3 Department of Medical Oncology, The First Affiliated Hospital of Guangxi Medical University, Nanning, Guangxi Zhuang Autonomous Region, China; 4 Department of Respiratory and Critical Care, The First Affiliated Hospital of Guangxi Medical University, Nanning, Guangxi Zhuang Autonomous Region, China

**Keywords:** Lung squamous cell carcinoma, MMP12, Gene expression, Prognosis, Clinical value, Immunity

## Abstract

**Background:**

Worldwide, lung squamous cell carcinoma (LUSC) has wreaked havoc on humanity. Matrix metallopeptidase 12 (*MMP12*) plays an essential role in a variety of cancers. This study aimed to reveal the expression, clinical significance, and potential molecular mechanisms of *MMP12* in LUSC.

**Methods:**

There were 2,738 messenger RNA (mRNA) samples from several multicenter databases used to detect *MMP12* expression in LUSC, and 125 tissue samples were validated by immunohistochemistry (IHC) experiments. Receiver operator characteristic (ROC) curves, Kaplan–Meier curves, and univariate and multivariate Cox regression analyses were used to assess the clinical value of *MMP12* in LUSC. The potential molecular mechanisms of *MMP12* were explored by gene enrichment analysis and immune correlation analysis. Furthermore, single-cell sequencing was used to determine the distribution of *MMP12* in multiple tumor microenvironment cells.

**Results:**

*MMP12* was significantly overexpressed at the mRNA level (*p* < 0.05, SMD = 3.13, 95% CI [2.51–3.75]), which was verified at the protein level (*p* < 0.001) by internal IHC experiments. *MMP12* expression could be used to differentiate LUSC samples from normal samples, and overexpression of *MMP12* itself implied a worse clinical prognosis and higher levels of immune cell infiltration in LUSC patients. *MMP12* was involved in cancer development and progression through two immune-related signaling pathways. The high expression of *MMP12* in LUSC might act as an antigen-presenting cell–associated tumor neoantigen and activate the body’s immune response.

**Conclusions:**

*MMP12* expression is upregulated in LUSC and high expression of *MMP12* serves as a risk factor for LUSC patients. *MMP12* may be involved in cancer development by participating in immune-related signaling pathways and elevating the level of immune cell infiltration.

## Introduction

Since cancer cells have multiple means of evading elimination by the immune system, there is still a shortage of effective cures for cancer. Lung cancer is one of the deadliest malignancies in the world and is the primary cause of death among cancer patients (Siegel, Miller & Jemal, 2020; Mridha et al., 2022; Adams et al., 2023). Lung squamous cell carcinoma (LUSC) belongs to non-small-cell lung cancer (NSCLC), and clinical statistics show that the number of patients with LUSC accounts for about 30% of patients with NSCLC (Li et al., 2022a; Ding et al., 2022; Chen et al., 2022a; Pan et al., 2022). In recent years, conventional platinum-based two-drug therapy and combination chemotherapy have remained the first-line treatments for advanced LUSC. However, due to aggressiveness, late diagnosis, and poor treatment response, LUSC poses a colossal treatment challenge, with an overall five-year survival rate of only about 18% for patients (Yang et al., 2022; Wang et al., 2022; Liu et al., 2022b; Huang et al., 2022; Ji et al., 2022; Xiao et al., 2022). Therefore, there is an urgent need to further explore the potential biomarkers of LUSC and provide a reliable basis for studying cancer immunotherapy targets.

Matrix metallopeptidase 12 (*MMP12*), also known as macrophage metalloelastase, is produced primarily by macrophages and is a member of the matrix metalloproteinase family. MMP12 is involved in various normal physiological functions, including the catabolism of the extracellular matrix and elastin degradation. It is also involved in the progress of various diseases, such as inflammatory lung diseases and arthritis (Nénan et al., 2005). Previously, a high expression of *MMP12* was found to be closely associated with the occurrence and progression of cancers at different sites, such as lung adenocarcinoma (LUAD) (Lv et al., 2015), colorectal cancer (Yu et al., 2021), hepatocellular carcinoma (Guo & Jiang, 2022), cervical cancer (Lin et al., 2021), and breast cancer (Cheng et al., 2021). In particular, *MMP12* expression is elevated in squamous carcinomas at other anatomical sites, such as esophageal squamous cell carcinoma (ESCC) (Mao et al., 2022) and oral squamous cell carcinoma (OSCC) (Chen et al., 2022b). *MMP12* promotes macrophage proliferation *via* the ERK/P38 MAPK pathway, and the knockdown of *MMP12* inhibits the development of some tumor cells (Lv et al., 2015). A growing body of evidence suggests a clear association between *MMP12* upregulation and the development and progression of multiple malignancies. Previous studies showed that *MMP12* expression was upregulated in 57 cases of LUSC tissues, and that high expression of *MMP12*, risk score, age, tumor stage, and TMN stage were significantly associated with prognosis of LUSC patients. However, the aforementioned studies have certain limitations (Ma et al., 2020; Zhang et al., 2020). For example, the studies were conducted based on small samples (only 720 samples from public databases), lacked internal sample validation, and did not separately analyze the immune mechanisms involved in *MMP12*. Therefore, further comprehensive investigation of the clinical value and mechanism of *MMP12* in LUSC is warranted.

In this study, we first determined the specific expression levels of *MMP12* in LUSC by analyzing large samples from multicenter and immunohistochemistry (IHC) results. Secondly, we focused on the potential molecular mechanisms and critical clinical implications of *MMP12* in LUSC. Finally, we extensively validated the significant correlation of *MMP12* expression with immune signaling pathways, immune cell levels, and immune cell infiltration. In conclusion, this study revealed, for the first time, the clinical significance and potential mechanisms of *MMP12* in LUSC, providing new insights into the possible association between *MMP12* and LUSC immune cells.

## Materials and Methods

### Data acquisition and data processing

The high-throughput data related to LUSC for this study were obtained from multiple publicly available databases: the Gene Expression Omnibus (GEO), ArrayExpress, The Cancer Genome Atlas (TCGA), and Genotype-Tissue Expression (GTEx) databases. Among them, microarray and RNA-Seq data could be obtained directly from the GEO database (http://www.ncbi.nlm.nih.gov/geo). Data for patients diagnosed with LUSC were downloaded from TCGA (http://xena.ucsc.edu/) and included RNA-Seq and clinical information (including clinical characteristics and survival data). A total of 33 criteria-compliant data sets with 2,738 samples, including 1,348 LUSC samples and 1,390 non-LUSC samples, were included in this study (Supplementary Material 1). In addition, we obtained four datasets (GSE37745, GSE29013, GSE30219, and GSE73403) involving a total of 221 LUSC cases to analyze whether *MMP12* expression was associated with overall survival (OS) in LUSC patients.

The “limma” package (Ritchie et al., 2015) was used to process the data sets from public databases and to normalize them using log_2_ (*x* + 1). Since the experimental batches differed for each data set, this study combined 33 LUSC cohorts into 10 new fellows based on the same platform. For example, GSE31552 and GSE44077 were merged into the reorganization cohort GPL6244 since both data sets were from the same platform: GPL6244. Finally, the “SVA” package (Leek & Storey, 2007) was used to eliminate batch effects.

### Evaluation of MMP12 protein expression in LUSC tissues based on internal IHC staining

To clarify the differences in MMP12 protein expression between LUSC and non-LUSC patients, we collected 125 tissue samples (106 LUSC samples; 19 control samples) from the First Affiliated Hospital of Guangxi Medical University. A total of 125 internal samples were divided into three tissue microarray sections (Nos. LUC481, LUC1021, and LUC1502, constructed by Guilin Fanpu Biotech, Guangxi Zhuang Autonomous Region, China) for further IHC experiments. The relevant operations were performed according to the manufacturer’s standards. First, tissue sections were fixed by formalin and embedded in paraffin, then sections were dewaxed, and antigen repair was performed with ethylenediaminetetraacetic acid buffer (pH = 9.0) at boiling state. After inactivation of endogenous enzyme activity, rabbit antihuman MMP12 monoclonal antibody (EPR11944[B], ab170414, dilution ratio 1:100) was added and stored overnight at 4 °C ambient. Next, a second antibody-labeled horseradish peroxidase (D-3004-15, Changdao Biotechnology Co., Ltd., Shanghai, China) was added to the tissue sections and incubated at 25 °C for 30 min. Subsequently, the slides were stained with diaminobenzidine, taking care to rinse the slides with PBS between steps. After hematoxylin restaining, the slides were dehydrated with a gradient concentration of ethanol solution and finally sealed with neutral gum. The sections were scored by microscopy, with brown representing positive and blue representing negative. The staining scores were judged as follows: 0 (no staining), 1 (mild staining), 2 (moderate staining), and 3 (severe staining). Different numbers of positive cells represented different scores: 0 (number <5%), 1 (5%–25%), 2 (26%–50%), 3 (51%–75%), and 4 (number > 75%). The total IHC score was generated by the product of the sample staining score and the percentage of positive cells, and the above assessments were performed independently by two senior pathologists. The raw data of IHC staining were presented in Supplementary Material 2. This study was approved and supported by the Medical Ethics Review Committee of the First Affiliated Hospital of Guangxi Medical University (2021 [KY-E-246]).

### *MMP12* expression and its clinical relevance in LUSC

The ability of *MMP12* expression to discriminate between tumor and control tissues could be determined using receiver operator characteristic (ROC) curves and summary ROC (sROC) curves based on sensitivity, specificity, and area under the curve (AUC) values. It was generally considered that larger sensitivity, specificity, and AUC values indicate a more remarkable ability of *MMP12* to differentiate between cancer and control tissues. Based on Yoden Index, true positive rate, false positive rate, true negative rate, false negative rate and cut-off values were calculated for each data set , and Stata (v15.0) was used to plot ROC curves.

In addition, this study further analyzed the association between *MMP12* expression and the survival outcomes of LUSC patients by establishing Kaplan–Meier curves and univariate and multivariate Cox regression analyses. The clinical indicators (gender, age, tumor stage, node stage, metastasis stage, clinical stage, and *MMP12*) and prognostic information utilized in the above analyses were available from the Xena database.

### *MMP12* potential mechanism in LUSC

Cistrome Data Browser (Zheng et al., 2019) contains a vast number of human transcription factors (TF), histone modifications, and chromatin accessibility samples. It is currently one of the most comprehensive chromatin immunoprecipitation sequence (ChIP-Seq)-related databases. To explore the potential molecular mechanism of *MMP12* in LUSC, this study predicted the TFs of target genes based on the Cistrome Data Browser and the standardized mean difference (SMD). In addition, ChIP-Seq data in the Cistrome Data Browser were used to validate our results and IGV (2.16.0) was used to visualize them. ChIP-Seq data must pass all quality tests and target gene score > 1. JASPAR (Fornes et al., 2020) is a free and publicly available TF database that focuses on collecting relevant TFs with DNA-binding site motifs. Therefore, the relevant TF motif data and the potential promoter region of *MMP12* in this study could be directly obtained from JASPAR and NCBI, respectively. Subsequently, the FIMO tool in the MEME suite was selected to mine the upstream transcription start site (TSS) of *MMP12* and the potential binding sequences of these motifs.

Gene Set Enrichment Analysis (GSEA) was performed using the “clusterProfiler” package (Wu et al., 2021), hoping to further explore the potential signaling pathways that *MMP12* may involve in LUSC. The signaling pathways shown in this analysis were obtained from the Kyoto Encyclopedia of Genes and Genomes database. To predict the relationship between the core genes of the enriched pathways, an interaction database platform, STRING (v11.0), was used to construct a protein–protein interaction (PPI) network (Crosara et al., 2018). This search was performed with “Homo sapiens” as the set species, the confidence score was set to 0.4, and other settings were set as default.

### Correlation of *MMP12* expression with the tumor microenvironment (TME)

Based on the TCGA-GTEx and GPL570 data sets, a single sample Gene Set Enrichment Analysis (ssGSEA) was used to analyze the differences in multiple immune cell scores between the high and low *MMP12* expression groups. Subsequently, we analyzed the correlation between *MMP12* and individual immune cell scores one by one and initially assessed the potential of *MMP12* for immune prediction.

The Tumor Immune Estimation Resource (TIMER) (Li et al., 2020; Chen et al., 2022c; Liu et al., 2022a; Li et al., 2022b) is an online database that allows comprehensive detection of immune cell infiltration levels. The data for six immune-infiltrating cells were downloaded from the database. The six immune cells included B cells, CD4+ T cells, CD8+ T cells, neutrophils, macrophages, and dendritic cells. In addition, to explore *MMP12* expression levels in various cells (*e.g.*, monocytes/macrophage), the Cancer Single-cell Expression Map (Zeng et al., 2022) was used to systematically investigate the heterogeneity of *MMP12* in different cells.

### Statistical analysis

The Wilcoxon rank-sum test and the SMD were used for comparisons between various groups. Among them, the Wilcoxon rank-sum test should be used when the combined data set was skewed and the variance of the totals to which the two samples belong was different. When the results show *I*^2^ values < 50% or chi-square test *p* - values > 0.05, there was less heterogeneity between the data sets, and we need to use a fixed-effects model for SMD calculations. The results of the SMD were statistically significant when the corresponding 95% confidence interval (CI) did not contain 0. The following criteria were used to determine differentially expressed genes: high expression: SMD > 0 and 95% CI excluding 0; low expression: SMD < 0 and 95% CI excluding 0. *Begg*’s test and *Egger*’s test evaluated the publication bias of SMD results, and *p* < 0.1 indicated a significant publication bias. Sensitivity analysis was used to evaluate the reliability of the analysis results. For hazard ratio (HR), the presence of a 95% CI of 1 or *p* > 0.05 indicated no statistical significance. In addition to the above statistical methods, the Spearman correlation coefficient was used to detect the correlation between *MMP12* and immune cells.

**Figure 1:**
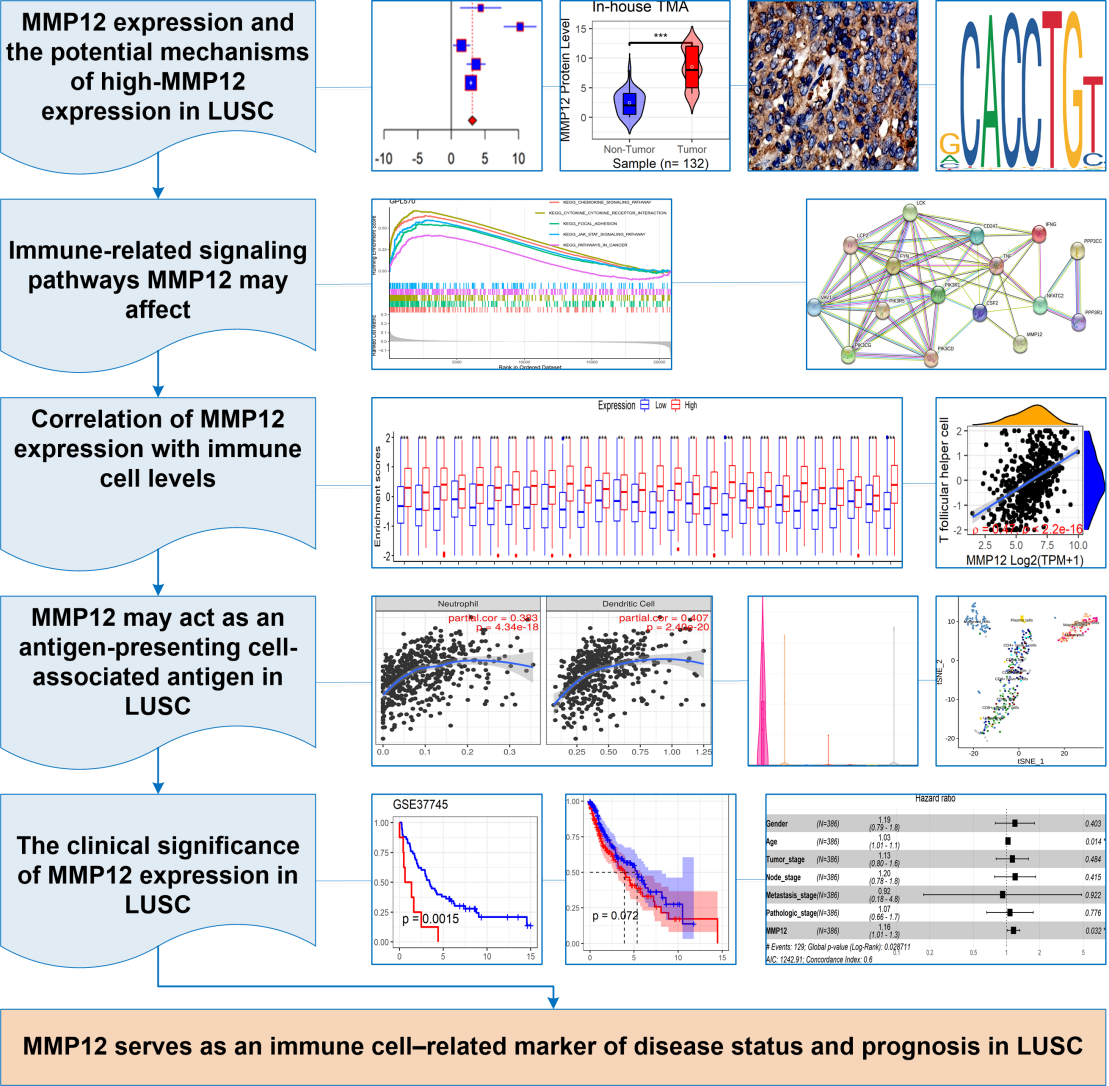
The research overflow of this study.

In this study, *p* < 0.05 represented a statistically significant difference. Stata (v15.0) was used for *Egger*’s tests, and sensitivity analysis, and R (v4.1.0) was used to complete all the remaining computational and visualization steps. The following was the research flow of this study (Fig. 1A).

## Results

### MMP12 expression level differences between LUSC and its control tissues

**Figure 2:**
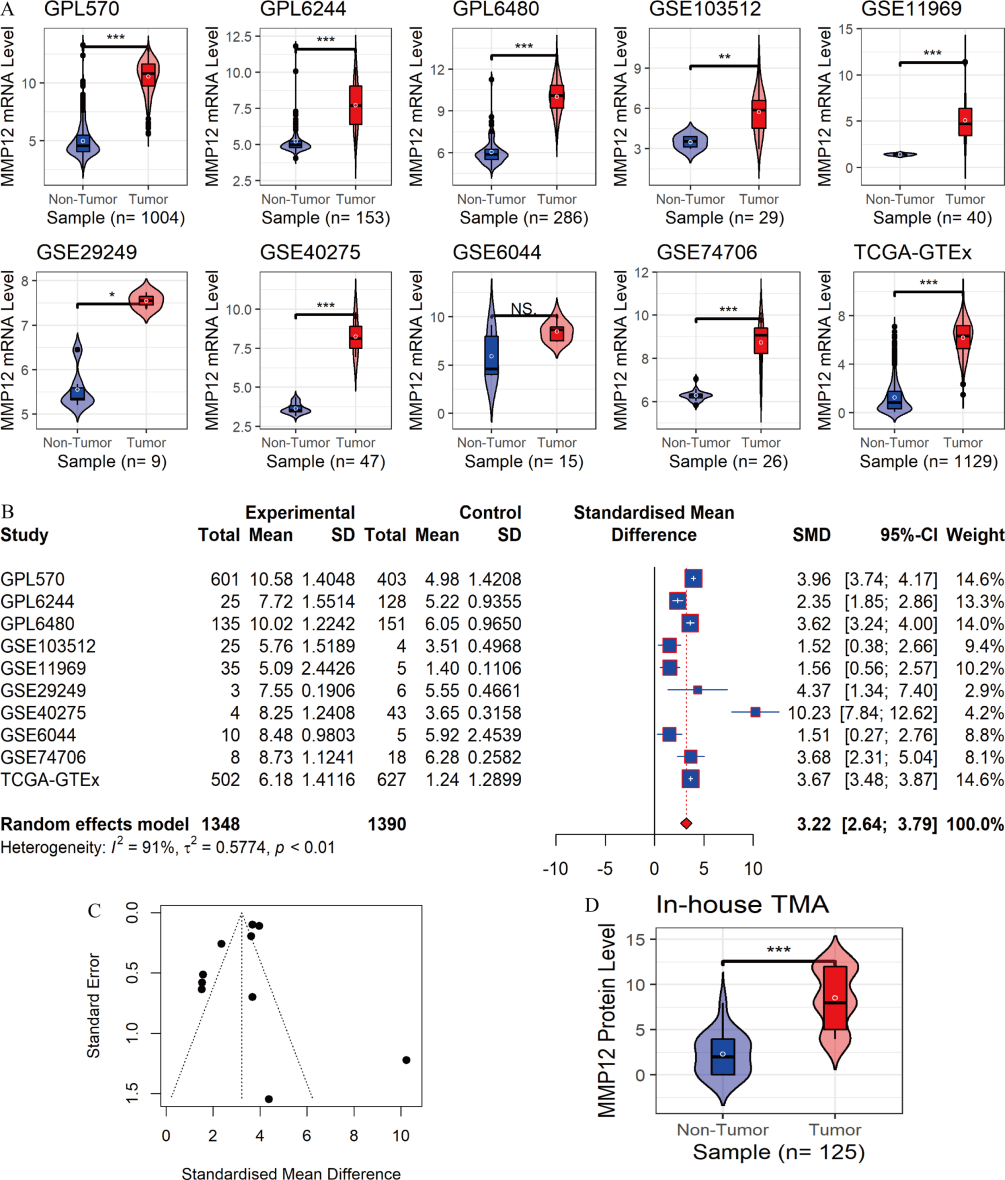
Differential expression levels of MMP12 between LUSC and its control tissues. (A) Violin plots of *MMP12* mRNA expression between LUSC and its control tissues in each data set. (B) Forest plot of *MMP12* mRNA expression between LUSC and its control tissues. (C) Funnel plot for detecting SMD publication bias. (D) Violin plot of MMP12 protein expression between LUSC and its control tissues. ^*ns*/*NS*^*p* > 0.05, ^∗^*p* < 0.05, ^∗∗^*p* < 0.01, ^∗∗∗^*p* < 0.001.

In the 10 data sets included in this study, statistically significant differences in *MMP12* expression between the LUSC group and control tissues were detected in nine cohorts (except for “GSE6044”). The results showed that *MMP12* mRNA expression levels were increased in the LUSC group in nine of the combined data sets mentioned above (*p* < 0.05; Fig. 2A). Then, a random-effects model showed that *MMP12* mRNA expression was elevated in the LUSC group but not in the control group (SMD = 3.13, 95% CI [2.51–3.75]; Fig. 2B), and no significant publication bias was detected (*p* > 0.1; Fig. 2C and Supplementary Material 3). Sensitivity analysis showed a small effect of low sample size on the overall *MMP12* expression in LUSC and a large heterogeneity of the data (Supplementary Material 4). We believed that the greater heterogeneity was related to the data coming from different experimental platforms, and a random-effects model was used in this study.

**Figure 3:**
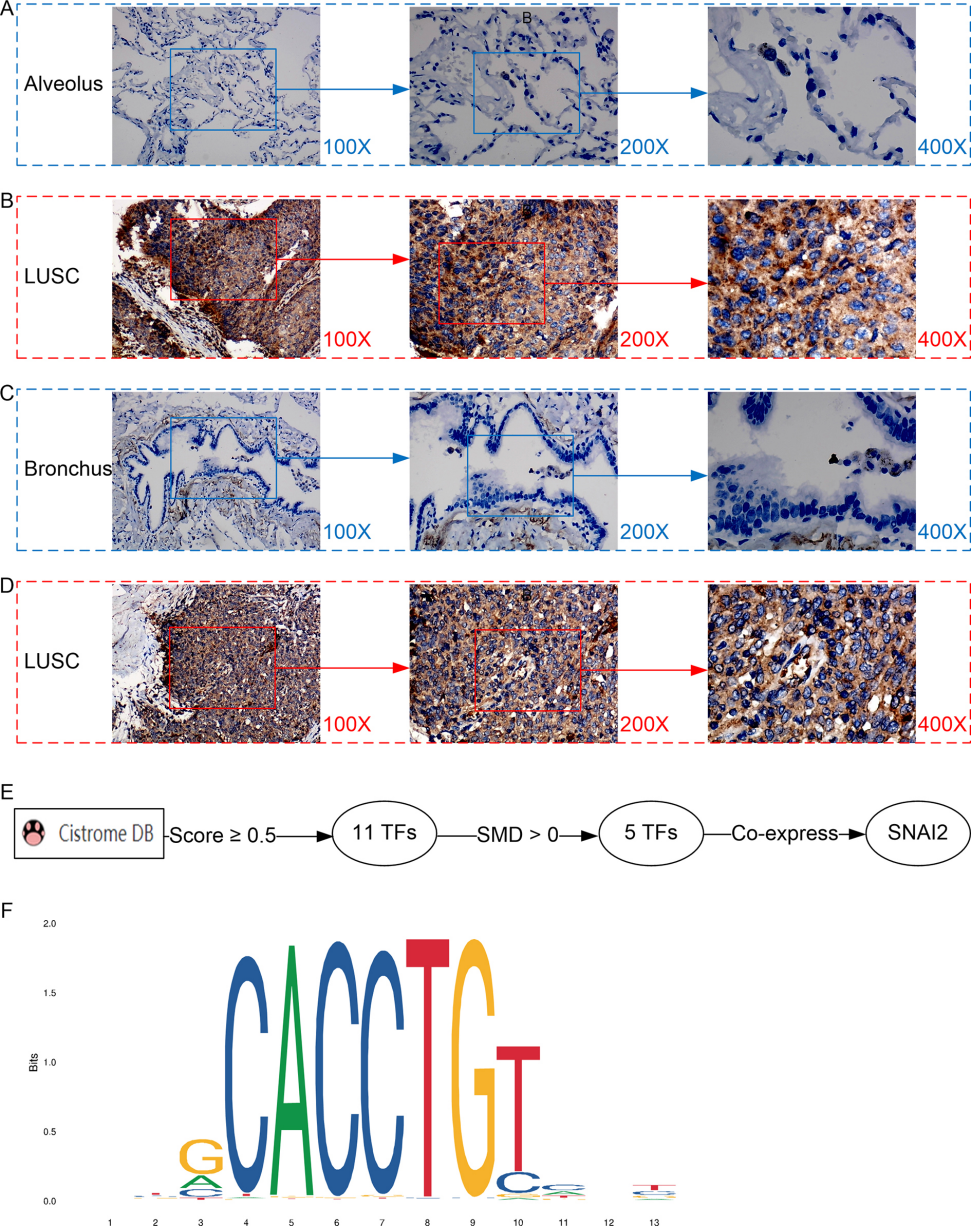
Protein expression and potential molecular mechanisms of MMP12 in LUSC. (A–D) Microscopic images showing anti-MMP12 antibodies exhibiting different levels of staining in alveolar, bronchial, and LUSC tissue. (E) After screening, *SNAI2* was identified as a possible transcription factor that regulated *MMP12* expression. (F) Motif map of *SNAI2*.

Furthermore, the expression level of MMP12 in LUSC was further verified using internal immunohistochemical experiments. Compared to the non-LUSC group, MMP12 protein levels were significantly upregulated in the LUSC group samples (*p* < 0.001; Fig. 2D), consistent with the mRNA expression levels. Further, under the microscope, as seen in Figs. 3A–3D, MMP12 protein could be easily observed in LUSC tissues (Figs. 3B and 3D) but not in control tissues (Figs. 3A and 3C).

### A potential molecular mechanism for the high expression of MMP12 in LUSC

As shown in the flow of Fig. 3E, based on the Cistrome Data Browser for calculation, we selected 11 TFs with a selection score greater than or equal to 0.5. Subsequently, the results of the SMD calculation and co-expression analysis determined that *SNAI2* was a potential TF regulating *MMP12* expression and positively correlated with *MMP12* expression. The peak signal of *SNAI2* ChIP-Seq was located within 2 kb upstream of *MMP12*, further validating the above conclusion (Supplementary Material 5).

The motif of *SNAI2* was shown in Fig. 3F. Combined with JASPAR and FIMO tools, we obtained the binding sequence of *SNAI2* upstream of *MMP12* TSS:GCACCTGTC. This study also identified two potential binding sites between the *SNAI2* motif and the TSS of *MMP12* (Supplementary Material 6).

### *MMP12* might affect immune-related signaling pathways

**Figure 4:**
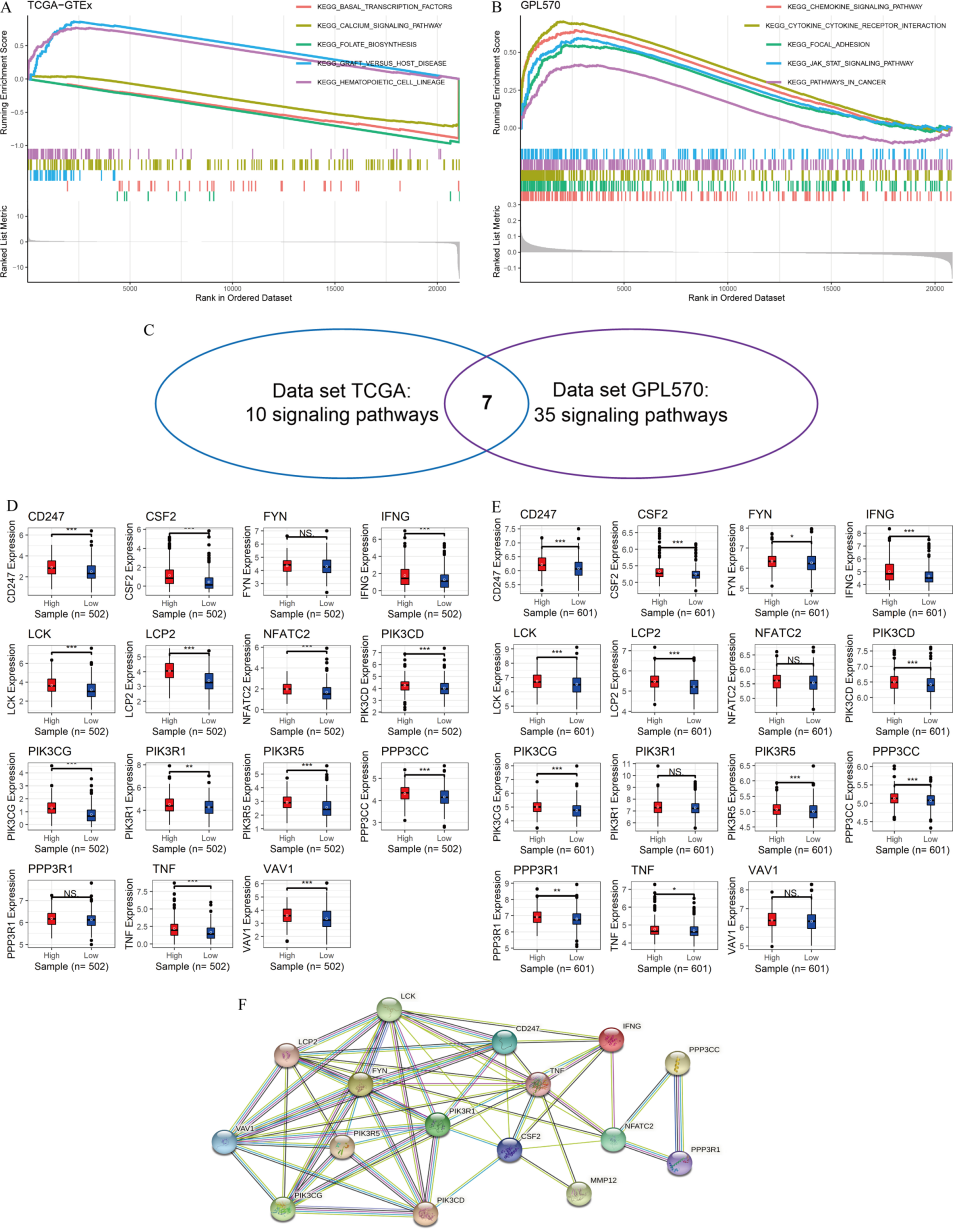
*MMP12* may affect immune-related signaling pathways. (A–C) LUSC samples based on data sets TGCA-GTEx (A) and GPL570 (B) *MMP12* might affect immune-related signaling pathways and their intersection (C). (D–E) LUSC samples based on data sets TGCA-GTEx (D) and GPL570 (E). LUSC samples: the expression differences of the core genes of the two pathways in the high and low *MMP12* expression groups. (F) Protein–protein interaction analysis of the core genes of the pathway. ^*ns*/*NS*^*p* > 0.05, ^∗^*p* < 0.05, ^∗∗^*p* < 0.01, ^∗∗∗^*p* < 0.001.

In this study, GSEA was used to explore the potential mechanisms of *MMP12* in multiple signaling pathways. The peaks of the curves appeared in the high-expression group, implying that these pathways were positively correlated with *MMP12* expression and that these signaling pathways became more active when *MMP12* expression was upregulated. Based on the LUSC samples from the data sets TCGA-GTEx (Fig. 4A) and GPL570 (Fig. 4B), GSEA suggested that *MMP12* was associated with multiple signaling pathways, and the results of the two data sets overlapped to obtain seven common pathways (Fig. 4C). Notably, two pathways (the T-cell receptor signaling pathway and the natural killer cell–mediated cytotoxicity) showed a direct association of *MMP12* with immune cells, suggesting that *MMP12* was likely to be involved in essential mechanisms of cancer through immune-related signaling pathways (Supplementary Material 7).

Subsequently, for the core genes enriched to both pathways, differential expression of most genes was observed between the high and low *MMP12* expression groups, and all of these genes were upregulated in the high *MMP12* expression group (*p* < 0.05; Figs. 4D and 4E). Finally, this study performed a PPI analysis of the core genes enriched in both pathways and found that these genes closely interacted with each other (Fig. 4F). The black and green connecting lines between *MMP12* and tumor necrosis factor (TNF) and *CSF2* suggested their co-expression and previous studies between the genes, respectively. We further explored previous studies finding that *MMP12* affects the activity of the *TNF* signaling pathway and monocyte-derived *MMP12* is induced by *CSF2* (Björnfot Holmström et al., 2017; Mao et al., 2022). Furthermore, we found that LCK proto-oncogene (LCK) had the highest number of PPI nodes with other genes, implying that *LCK* might be the central gene in both signaling pathways and had multiple different interactions with other genes, followed by *PIK3R1* and *TNF*.

### *MMP12* positively correlated with immune cell levels

**Figure 5:**
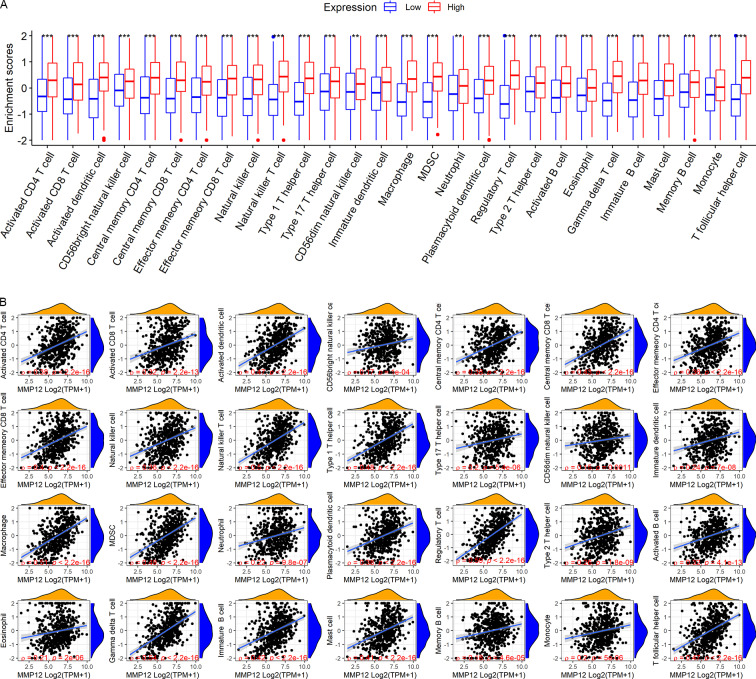
Based on LUSC samples from the TCGA-GTEx data set, the correlation between *MMP12* and immune cell levels. (A) Different immune cell scores between the high and low *MMP12* expression groups. (B) Correlation between *MMP12* and immune cell scores. ^∗^*p* < 0.05, ^∗∗^*p* < 0.01, ^∗∗∗^*p* < 0.001.

**Figure 6:**
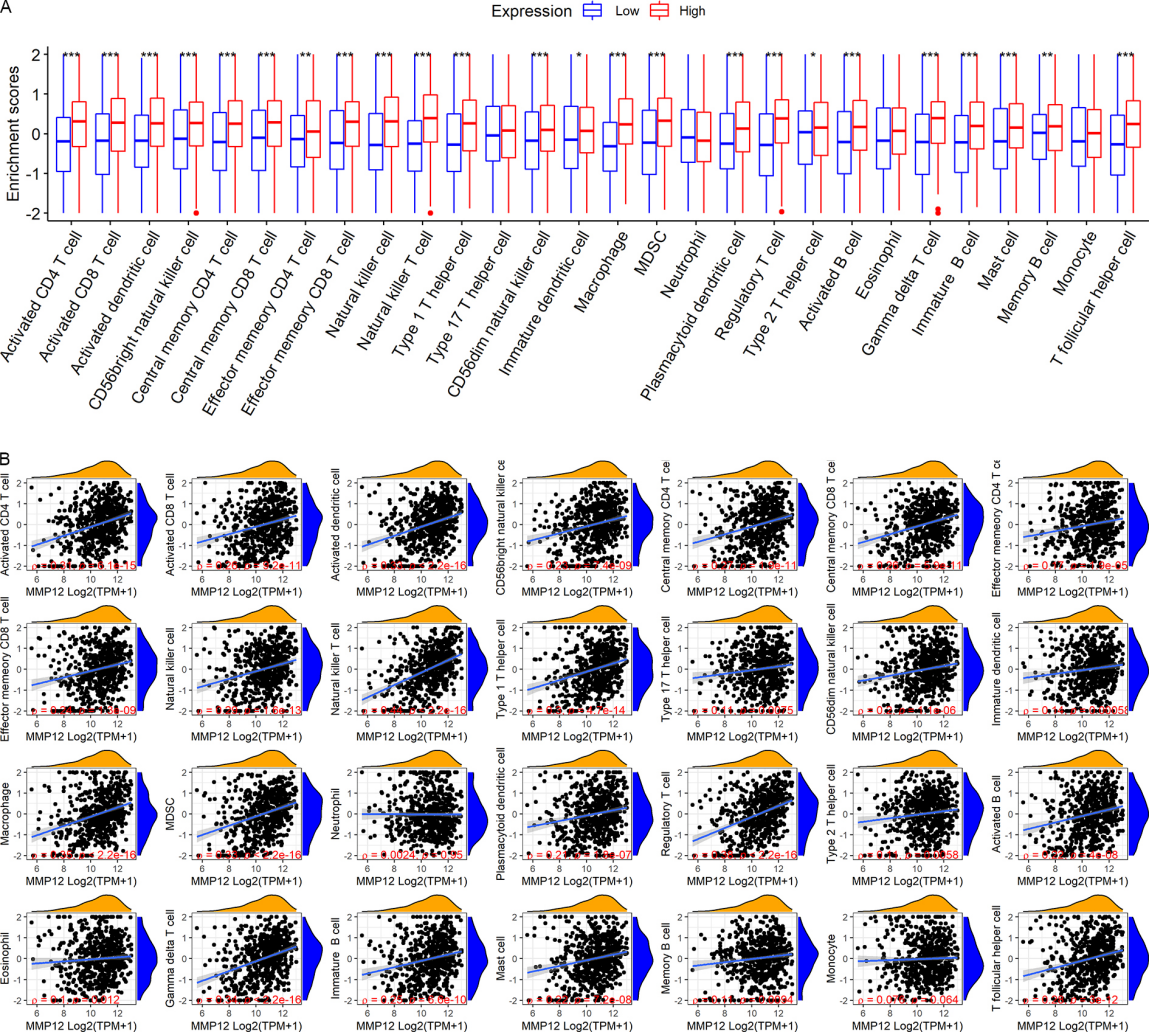
Based on LUSC samples from the GPL570 data set, the correlation between *MMP12* and immune cell levels. (A) Different immune cell scores between the high and low *MMP12* expression groups. (B) Correlation between *MMP12* and immune cell scores. ^∗^*p* < 0.05, ^∗∗^*p* < 0.01, ^∗∗∗^*p* < 0.001.

LUSC patients in the TCGA-GTEx cohort were divided into high and low *MMP12* expression groups using median *MMP12* expression. The correlation between *MMP12* expression and 28 immune cells was investigated using ssGSEA. The results showed significant differences in immune cell scores between the high and low *MMP12* expression groups, and higher levels of immune cells could be observed in the high *MMP12* expression group (*p* < 0.01; Fig. 5A). Considering that the association between them could still not be intuitively determined by difference analysis alone, we further explored the correlation between *MMP12* and these immune cell scores. As can be found in Fig. 5B, the expression level of *MMP12* tended to be positively correlated with the immune cell score (*ρ* > 0.15, *p* < 0.01). Most of these results were validated by the GPL570 data set (Figs. 6A and 6B).

### *MMP12* might serve as an antigen-presenting cell–associated LUSC antigen

**Figure 7:**
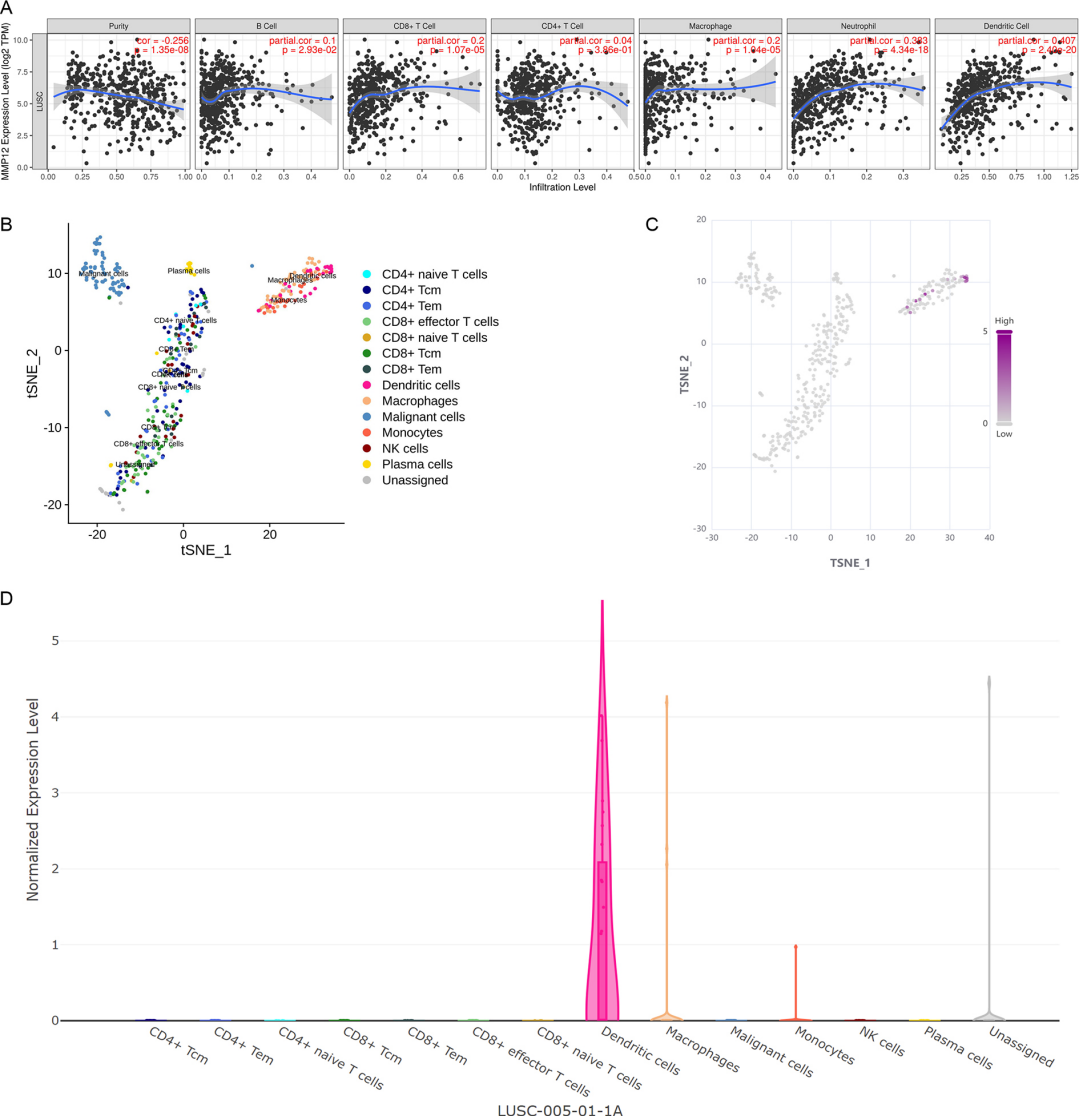
MMP12 might play a role as an antigen-presenting cell–associated LUSC antigen. (A) *MMP12* was closely associated with antigen-presenting cells. (B) *MMP12* was expressed at high levels in immune cells. (C) *MMP12* was highly expressed in antigen-presenting cells. (D) The expression of *MMP12* was significantly increased in DC and monocytes/macrophages.

Considering that the immune response plays a crucial role in antitumor, this study further verified the potential association between *MMP12* expression and immune cells in TME using different methods. Based on the TIMER algorithm (Fig. 7A), we found that *MMP12* expression was significantly negatively correlated with tumor purity (*ρ* < 0, *p* < 0.001) and that upregulation of *MMP12* expression was associated with increased infiltration levels of five immune cell types (except CD4+ T cells) (*ρ* > 0, *p* < 0.05). From the previous results, it was clear that *MMP12* in LUSC was positively correlated not only with the level of specific immune cells (*e.g.*, CD 8 + T cells) but also with the level of non-specific immune cell infiltration (*e.g.*, neutrophils), suggesting that it might have the potential to activate the immune system.

Further analysis showed that multicenter single-cell sequencing data results also suggested that *MMP12* expression was evident in immune cells in several classes of TME-related cells, such as dendritic cells (DC) and monocytes/macrophages (Figs. 7B–7D and Supplementary Material 8). This suggested that the high expression of *MMP12* in LUSC might act as an antigen-presenting cell–associated tumor neoantigen and activate the body’s immune response.

### *MMP12* discriminated between LUSC tissues and normal lung tissues

**Figure 8:**
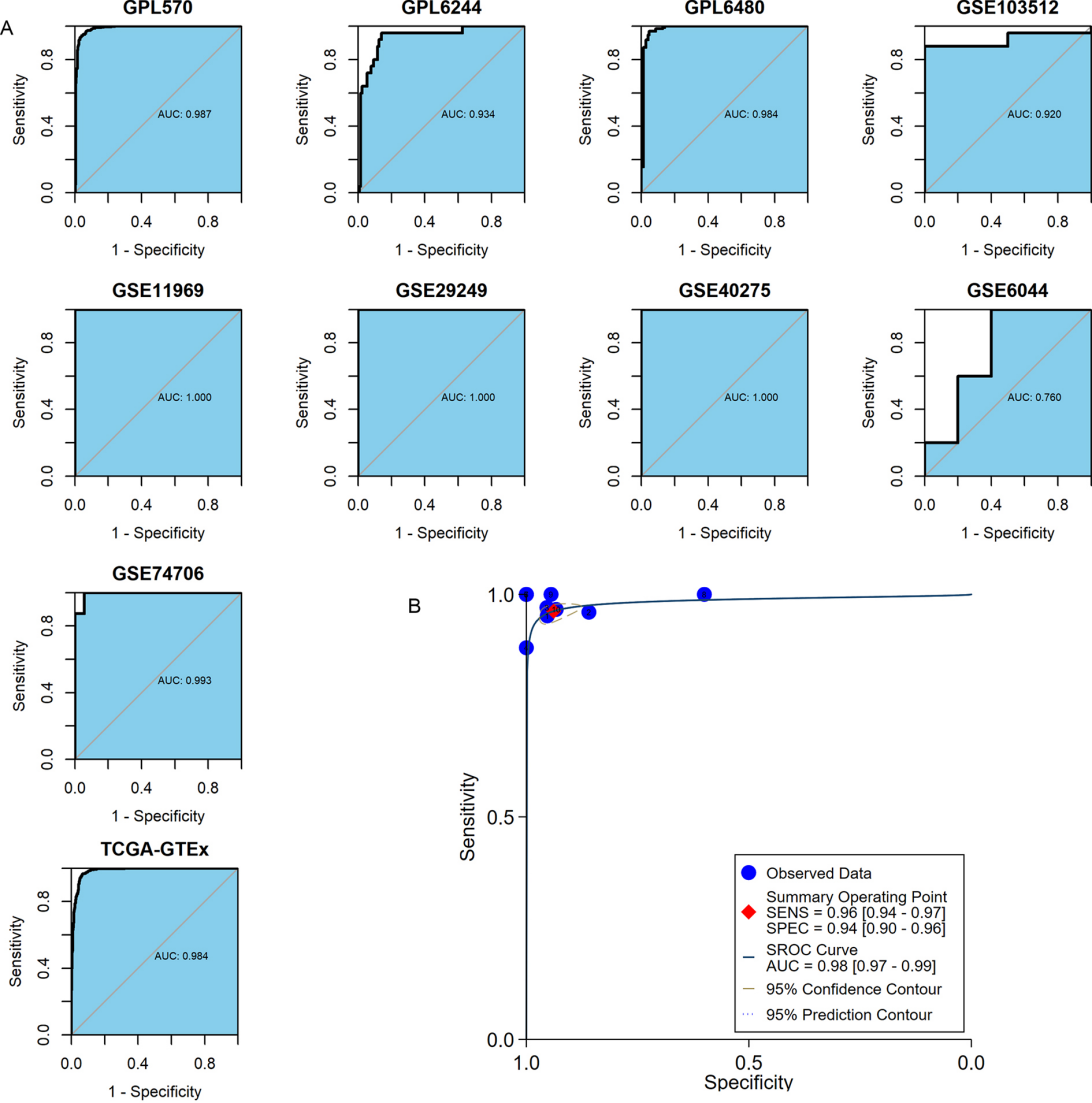
The ability of *MMP12* to distinguish cancerous tissues from control tissues. (A) Receiver operating characteristic curves. (B) Summary receiver operating characteristic curve.

The important clinical value of *MMP12* in TME has already been observed, so this study expected that *MMP12* could further demonstrate a valuable clinical role in LUSC by ROC and sROC curves. In the included 10 combined data sets, *MMP12* mRNA expression possessed a very high precision to distinguish LUSC samples from control samples according to the ROC curves of nine of the cohorts (AUC > 0.9) (Fig. 8A). The sROC analysis showed that *MMP12* expression made it feasible to distinguish LUSC samples from control tissue samples (sensitivity = 0.96, specificity = 0.94, AUC = 0.98) (Fig. 8B). These results suggested that *MMP12* had significant potential in the identification of LUSC.

### Correlation of *MMP12* with the prognosis of LUSC patients

**Figure 9:**
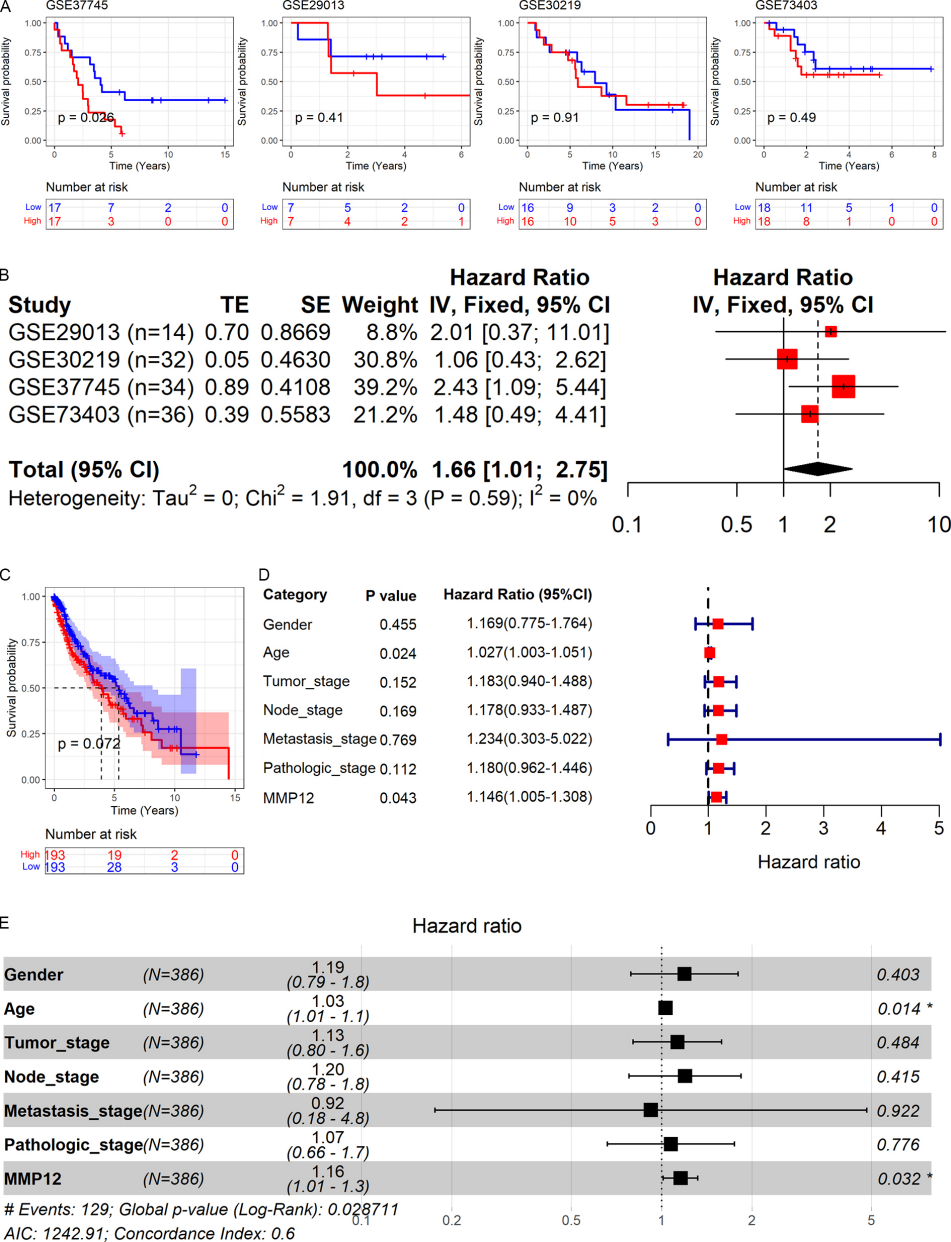
Correlation of *MMP12* with the prognosis of LUSC patients. (A) Kaplan–Meier curve for GEO data. (B) Univariate Cox regression analysis for the GEO data. (C) Kaplan–Meier curve for TCGA data. (D) Univariate Cox regression analysis for TCGA data. (E) Multivariate Cox regression analysis of TCGA data.

As shown above, *MMP12* might be a potential marker for the identification of LUSC. Therefore, based on GEO and TCGA data, this study explored the relationship between *MMP12* expression and the prognosis of LUSC patients. As shown in Figs. 9A and 9C, high *MMP12* mRNA expression was associated with poor OS, and the OS of LUSC patients with *MMP12* overexpression was poorer. A trend showed that overexpressed *MMP12* could be a prognostic risk factor for LUSC patients, and only results based on the GSE37745 cohort were statistically significant (*p* = 0.026). Subsequently, the four GEO data sets were further combined and it was also demonstrated by prognostic analysis that overexpression of *MMP12* leads to poorer prognosis in LUSC patients (Fig. 9B). The four GEO data sets covered 116 samples; compared to patients with reduced *MMP12* expression, patients with elevated *MMP12* expression tended to have a poorer OS (HR = 1.66, 95% CI [1.01–2.75]).

Further, the effect of different clinical indicators (gender, age, tumor stage, node stage, metastasis stage, pathologic stage, and *MMP12* expression level/value on the OS of LUSC patients was investigated by establishing Cox regression analysis. The results showed that both increased age and upregulated MMP12 protein expression resulted in more adverse OS in LUSC patients (*p* < 0.05; Fig. 9D), and the multivariate Cox regression analysis showed similar results (*p* < 0.05; Fig. 9E).

## Discussion

To our knowledge, a comprehensive study on *MMP12* in LUSC has not previously appeared; this paper fills this part of the gap in several aspects, including differential expression, potential molecular mechanisms, immune relevance, and clinical significance. In this study, 1,348 LUSC samples and 1,390 control samples were collected for a comprehensive analysis, which showed elevated levels of *MMP12* mRNA in LUSC. The IHC staining results suggested that MMP12 expression was elevated in LUSC tumor cells at the protein level, and this result was supported by Ella et al. (2018). *MMP12* overexpression in LUSC exhibited significant prognostic and predictive values. Furthermore, *MMP12* might participate in immune-related signaling pathways and was closely associated with immune-infiltrating cells in TME, implying that this gene might play a critical role in immune response.

The differential expression of *MMP12* in cancer has received much attention, and its high expression has been reported several times. Elshimi et al. (2019) observed that MMP12 was involved in tumor aggressiveness and metastasis and that overexpressed blood mRNA *MMP12* could be used for the early diagnosis of hepatocellular carcinoma. Lin et al. (2021) also found that *MMP12* was highly expressed in cervical cancer and that inhibition of *MMP12* expression could significantly reduce the metastasis of cervical cancer cells. Ella et al. (2018) found that *MMP12* produced by tumor cells promoted proliferation of lung tumors. Meanwhile, previous studies have shown that high expression of *MMP12* is closely associated with poor prognosis in ESCC and OSCC. Therefore, we focused our attention on squamous tumors in the lung. In LUSC, Zhang et al. (2020) only mentioned *MMP12* differentially expressed in this disease; however, the research was based on limited samples (*n* of samples = 620), and *MMP12* was not explored deeply enough in LUSC. In our study, upregulation of *MMP12* expression in multiple cancer data sets (*n* of samples = 2738) was detected using differential analysis and SMD. Furthermore, we validated this result at the protein level using in-house IHC experiments (*n* of samples = 125), with tissue samples originating from the First Affiliated Hospital of Guangxi Medical University. These new attempts exemplified the strengths of this study.

Although dysregulation of *MMP12* in cancer is common, its underlying molecular mechanisms remain unclear. This study provided a basis for revealing the underlying molecular mechanisms of *MMP12*. Through the computation and screening of the Cistrome Data Browser, *SNAI2* was determined as a potential TF regulating the upregulation of *MMP12* expression in LUSC. Previous studies have shown that *SNAI2* can induce the invasiveness of cancer cells (Fan et al., 2020); predictably, *MMP12* might be involved in LUSC metastasis as a downstream gene of *SNAI2*, and this proposition was reinforced by ChIP-Seq data in the Cistrome Data Browser. To the best of our knowledge, this finding has never been reported before, reflecting this study’s novelty. Subsequently, based on the LUSC samples from the TCGA-GTEx and GPL570 data sets, we found two signaling pathways showing a direct association of *MMP12* with immune cells (the T-cell receptor signaling pathway and natural killer cell–mediated cytotoxicity). This result focused our attention on the relevance of *MMP12* to immune cells in TME. Furthermore, the leading genes in the two signaling pathways were upregulated in the high-*MMP12* expression group. This result suggested that *MMP12* might be involved in the underlying mechanisms of cancer through immune-related signaling pathways. Interestingly, based on the results of PPI analysis, *LCK* was identified as a key gene in this network, implying that *LCK* played a crucial role in the immune-related pathways involved in *MMP12*. A study showed that the LCK protein is a constitutive component of the T-cell receptor (TCR) (Jury et al., 2003). LCK can regulate the TCR’s function to recognize pathogens’ antigens and generate immune responses (Cai et al., 2020). Based on interconnected cues, we inferred that *MMP12* was further involved in the underlying mechanisms of LUSC by participating in T lymphocyte-dominated adaptive reactions.

Since the previous results show that *MMP12* is involved in the potential mechanisms of cancer through immune-related signaling pathways, we focused on the cells associated with TME. Immune cells account for a large proportion of TME (Wu et al., 2019). *MMP12* was significantly and positively correlated with immune cell levels, and immune cell scores were higher in the *MMP12* high-expression group, suggesting that *MMP12* was very closely associated with immune cells. We further validated this using the TIMER algorithm (Chen et al., 2022c; Li et al., 2020; Li et al., 2022b; Liu et al., 2022a) and multicenter single-cell sequencing, and the results were mostly consistent with our expectations; upregulation of *MMP12* expression was not only associated with increased infiltration levels of five immune cell types but was also widely present in DCs as well as monocytes/macrophages. DCs are involved in antitumor immunity by activating T cells, and macrophages can participate in immune regulation through phagocytosis, antigen presentation, and the secretion of cytokines (Wynn & Vannella, 2016). MMP12 in DCs and macrophages plays an essential role in the processes of many diseases. For treating allergic rhinitis, Zhou et al. (2022) speculated that MMP12 might be a biomarker reflecting DC activity. Weng et al. (2018) found that MMP12 secreted by M2-type macrophages was involved in the regression of liver fibrosis. Furthermore, the close association of *MMP12* with immune cells suggests its possible involvement in tumorigenesis and development. For example, inflammatory cytokines activate mesenchymal stromal cells in breast cancer and induce elevated levels of *MMP12* expression by neutrophils in TME (Yu et al., 2017). *MMP12* can affect ESCC-related pathway activity (*e.g.*, Toll-like receptor signaling pathway, TNF signaling pathway, and IL-17 signaling pathway), and is closely associated with the dysregulation of macrophage M0, B cells, eosinophils, and mast cells (activated or quiescent) (Mao et al., 2022). Overall, this study found that *MMP12* was highly expressed not only in tumor cells but also in some APCs of LUSC. *MMP12* possessed the potential to activate the body’s immune response as an antigen-presenting cell–associated tumor neoantigen, suggesting that *MMP12* might be a potential new immunotherapeutic target in LUSC.

Considering the tantalizing clinical significance of elevated *MMP12* expression in LUSC, this study attempted to provide valuable findings. *MMP12* expression could distinguish LUSC with high accuracy and thus demonstrated an excellent potential as a diagnostic biomarker. Multiple assays have shown that elevated *MMP12* expression in LUSC was associated with shorter survival time, and Cox regression analysis further revealed that age was also a significant factor in the shortened OS of LUSC patients. As mentioned above, the upregulation of *MMP12* expression can promote the growth and migration of various tumors (including LUSC) (Elshimi et al., 2019; Xu et al., 2018); however, *MMP12*′*s* protective effect on tumors has also been reported (Liu et al., 2021). This difference may result from several factors, including differences in tumor types and stages of development, or even different cells that express *MMP12*. For example, *MMP12* from vulvar squamous cell carcinoma possessed a more aggressive profile; *MMP12* levels were higher in well-differentiated grade I tumors (produced by macrophages) compared to grade III tumors (Decock et al., 2011).

There were still some limitations in this study: (1) We could not secure a sufficient number of samples for analyzing the differential expression of MMP12 at the mRNA and protein levels and its correlation with prognosis. (2) Although we explored the potential mechanism of *MMP12* action in LUSC in the signaling pathway and upstream TF, more in-depth studies were not conducted. (3) More *in vivo* and *in vitro* experiments should be added to further investigate and demonstrate *MMP12* as a potential biomarker of immune cells in LUSC.

## Conclusion

In conclusion, this study provides a comprehensive assessment of *MMP12* and determines that *MMP12* expression is upregulated in LUSC. High expression of *MMP12* serves as a risk factor for LUSC patients and may be an immune-related predictive and prognostic marker for LUSC. *MMP12* may be involved in cancer development by participating in immune-related signaling pathways and elevating the level of immune cell infiltration. In addition, *MMP12* is significantly expressed in DCs and monocytes/macrophages, which may provide new directions for the development of future immunotherapy for LUSC.

##  Supplemental Information

10.7717/peerj.15598/supp-1 Supplemental Information 1 MMP12 mRNA-related microarrays and RNA-Seq datasets included in this study

10.7717/peerj.15598/supp-2 Supplemental Information 2 Raw data from internal IHC staining

10.7717/peerj.15598/supp-3 Supplemental Information 3 The *egger* test shows no significant publication bias in the SMD results

10.7717/peerj.15598/supp-4 Supplemental Information 4 Sensitivity analysis suggested significant heterogeneity and a random effects model was used in this study to reduce the effect of heterogeneity

10.7717/peerj.15598/supp-5 Supplemental Information 5 SNAI2 ChIP-Seq analysis suggested a significant peak signal upstream of *MMP12*

10.7717/peerj.15598/supp-6 Supplemental Information 6 There are two potential biding sites between SNAI2 motif and the underlying promoter region (upstream 1 kb) of *MMP12*

10.7717/peerj.15598/supp-7 Supplemental Information 7 Based on the TCGA-GTEx and GPL570 datasets, *MMP12* was enriched in 10 and 35 signaling pathways, respectively

10.7717/peerj.15598/supp-8 Supplemental Information 8 *MMP12* expression was evident in immune cells in several classes of TME-related cells, such as dendritic cells (DC) and monocytes/macrophages

10.7717/peerj.15598/supp-9 Supplemental Information 9 Photographs of normal alveolar tissue under immunohistochemical staining at 100x microscopy

10.7717/peerj.15598/supp-10 Supplemental Information 10 Photographs of normal alveolar tissue under immunohistochemical staining at 200x microscopy

10.7717/peerj.15598/supp-11 Supplemental Information 11 Photographs of normal alveolar tissue under immunohistochemical staining at 400x microscopy

10.7717/peerj.15598/supp-12 Supplemental Information 12 Photographs of lung squamous cell carcinoma (LUSC) tissue (sample number 1) under immunohistochemical staining at 100x microscopy

10.7717/peerj.15598/supp-13 Supplemental Information 13 Photographs of lung squamous cell carcinoma (LUSC) tissue (sample number 1) under immunohistochemical staining at 200x microscopy

10.7717/peerj.15598/supp-14 Supplemental Information 14 Photographs of lung squamous cell carcinoma (LUSC) tissue (sample number 1) under immunohistochemical staining at 400x microscopy

10.7717/peerj.15598/supp-15 Supplemental Information 15 Photographs of normal bronchial tissue under immunohistochemical staining at 100x microscopy

10.7717/peerj.15598/supp-16 Supplemental Information 16 Photographs of normal bronchial tissue under immunohistochemical staining at 200x microscopy

10.7717/peerj.15598/supp-17 Supplemental Information 17 Photographs of normal bronchial tissue under immunohistochemical staining at 400x microscopy

10.7717/peerj.15598/supp-18 Supplemental Information 18 Photographs of lung squamous cell carcinoma (LUSC) tissue (sample number 2) under immunohistochemical staining at 100x microscopy

10.7717/peerj.15598/supp-19 Supplemental Information 19 Photographs of lung squamous cell carcinoma (LUSC) tissue (sample number 2) under immunohistochemical staining at 200x microscopy

10.7717/peerj.15598/supp-20 Supplemental Information 20 Photographs of lung squamous cell carcinoma (LUSC) tissue (sample number 2) under immunohistochemical staining at 400x microscopy

## Abbreviations

LUSC: lung squamous cell carcinoma
NSCLC: non-small-cell lung cancer
MMP12: Matrix metallopeptidase 12
LUAD: lung adenocarcinoma
mRNA: messenger RNA
GEO: the Gene Expression Omnibus
TCGA: The Cancer Genome Atlas
GTEx: Genotype-Tissue Expression
IHC: immunohistochemistry
ROC: receiver operator characteristic
AUC: area under the curve
TF: transcription factor
ChIP-Seq: chromatin immunoprecipitation sequence
SMD: standardized mean difference
TSS: transcription start site
GSEA: gene set enrichment analysis
PPI: protein-protein interaction
TME: tumor microenvironment
TIMER: The Tumor Immune Estimation Resource
CI: confidence interval
HR: Hazard Ratio
LCK: LCK proto-oncogene
DC: dendritic cells
OS: overall survival
TCR: T cell receptor

## Additional Information and Declarations

### Data Availability

The following information was supplied regarding data availability: The data is available at TCGA, LUSC (https://portal.gdc.cancer.gov/projects/TCGA-LUSC) and NCBI GEO: GSE29249, GSE103512, GSE74706, GSE40275, GSE6044, GSE11969, GSE19188, GSE19804, GSE30219, GSE18842, GSE101929, GSE157010, GSE106937, GSE18385, GSE33532, GSE50081, GSE43580, GSE37745, GSE28571, GSE29013, GSE10245, GSE2109, GSE27556, GSE31552, GSE44077, GSE51852, GSE33479, GSE101420, GSE73403, GSE40074, GSE40588.
