# Supplementary material for: MMP12 serves as an immune cell–related marker of disease status and prognosis in lung squamous cell carcinoma
Source: PeerJ. 2023 Aug 16;11:e15598. doi: 10.7717/peerj.15598 (PMC10439720; doi:10.7717/peerj.15598)
Supplement: Supplemental Information 1 [file peerj-11-15598-s001.docx]

**Supplementary material 1.** *MMP12* mRNA-related microarrays and RNA-Seq datasets included in this study.

| Platform | Dataset | LUSC sample | Non-LUSC sample |
| --- | --- | --- | --- |
| GPL10558 | GSE29249 | 3 | 6 |
| GPL13158 | GSE103512 | 25 | 4 |
| GPL13497 | GSE74706 | 8 | 18 |
| GPL15974 | GSE40275 | 4 | 43 |
| GPL201 | GSE6044 | 10 | 5 |
| GPL7015 | GSE11969 | 35 | 5 |
| GPL570 | GSE19188 | 27 | 65 |
| GPL570 | GSE19804 | 0 | 60 |
| GPL570 | GSE30219 | 61 | 14 |
| GPL570 | GSE18842 | 0 | 45 |
| GPL570 | GSE101929 | 0 | 34 |
| GPL570 | GSE157010 | 235 | 0 |
| GPL570 | GSE106937 | 1 | 4 |
| GPL570 | GSE18385 | 0 | 161 |
| GPL570 | GSE33532 | 16 | 20 |
| GPL570 | GSE50081 | 43 | 0 |
| GPL570 | GSE43580 | 73 | 0 |
| GPL570 | GSE37745 | 66 | 0 |
| GPL570 | GSE28571 | 28 | 0 |
| GPL570 | GSE29013 | 25 | 0 |
| GPL570 | GSE10245 | 18 | 0 |
| GPL570 | GSE2109 | 6 | 0 |
| GPL570 | GSE27556 | 2 | 0 |
| GPL6244 | GSE31552 | 25 | 62 |
| GPL6244 | GSE44077 | 0 | 66 |
| GPL6480 | GSE51852 | 28 | 4 |
| GPL6480 | GSE33479 | 14 | 27 |
| GPL6480 | GSE101420 | 0 | 60 |
| GPL6480 | GSE73403 | 69 | 0 |
| GPL6480 | GSE40074 | 24 | 0 |
| GPL6480 | GSE40588 | 0 | 60 |
| / | TCGA | 502 | 49 |
| / | GTEx | 0 | 578 |
| / | Total | 1348 | 1390 |
