# Supplementary figures and images for: MMP12 serves as an immune cell–related marker of disease status and prognosis in lung squamous cell carcinoma
Source: PeerJ. 2023 Aug 16;11:e15598. doi: 10.7717/peerj.15598 (PMC10439720; doi:10.7717/peerj.15598)

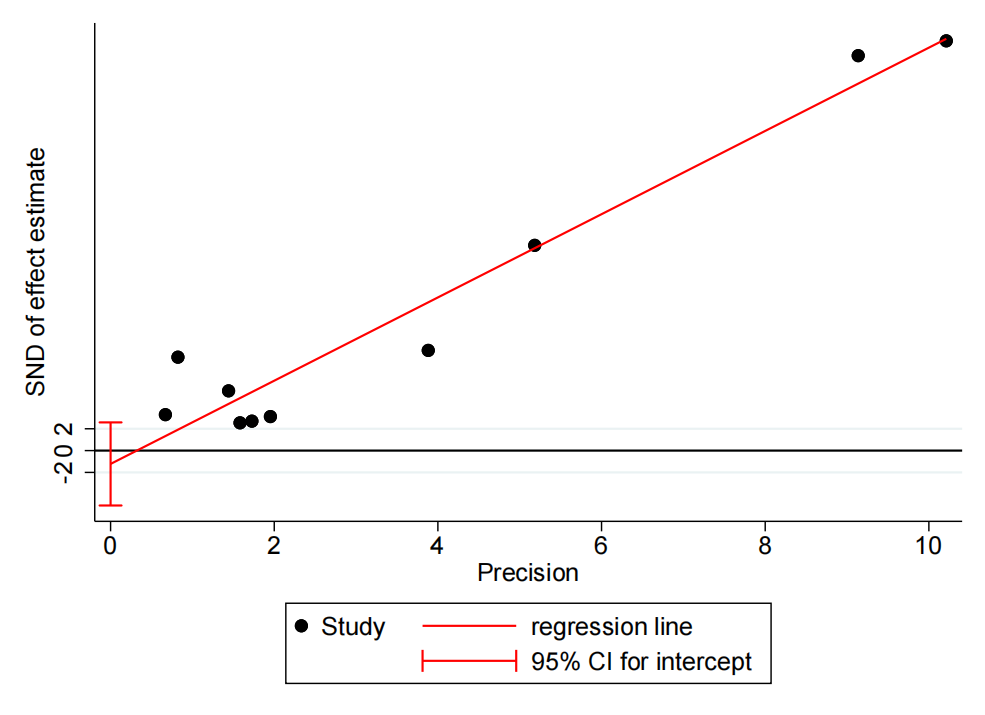

Supplement: Supplemental Information 3 [file peerj-11-15598-s003.png]

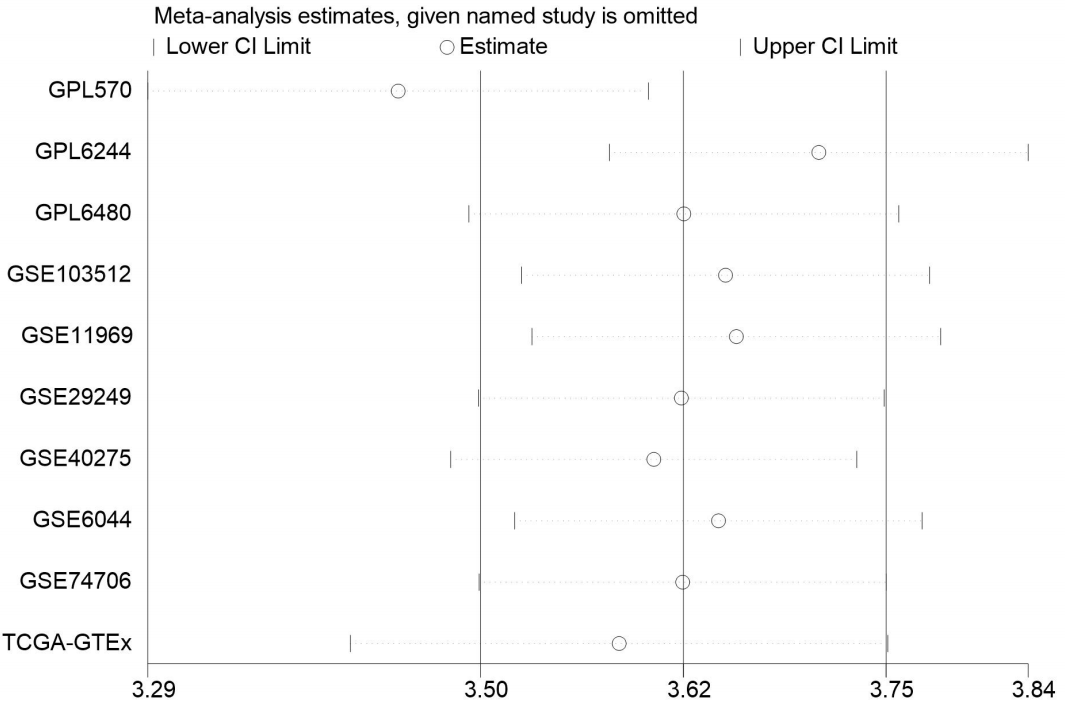

Supplement: Supplemental Information 4 [file peerj-11-15598-s004.png]

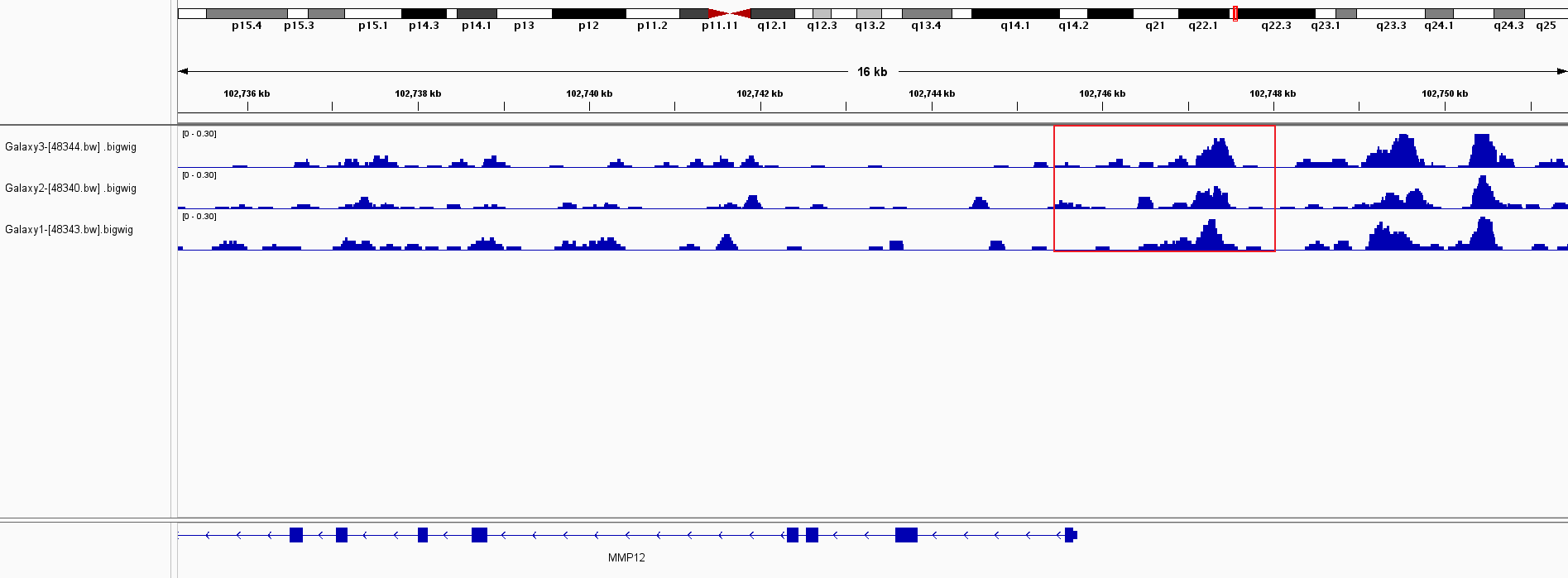

Supplement: Supplemental Information 5 [file peerj-11-15598-s005.png]

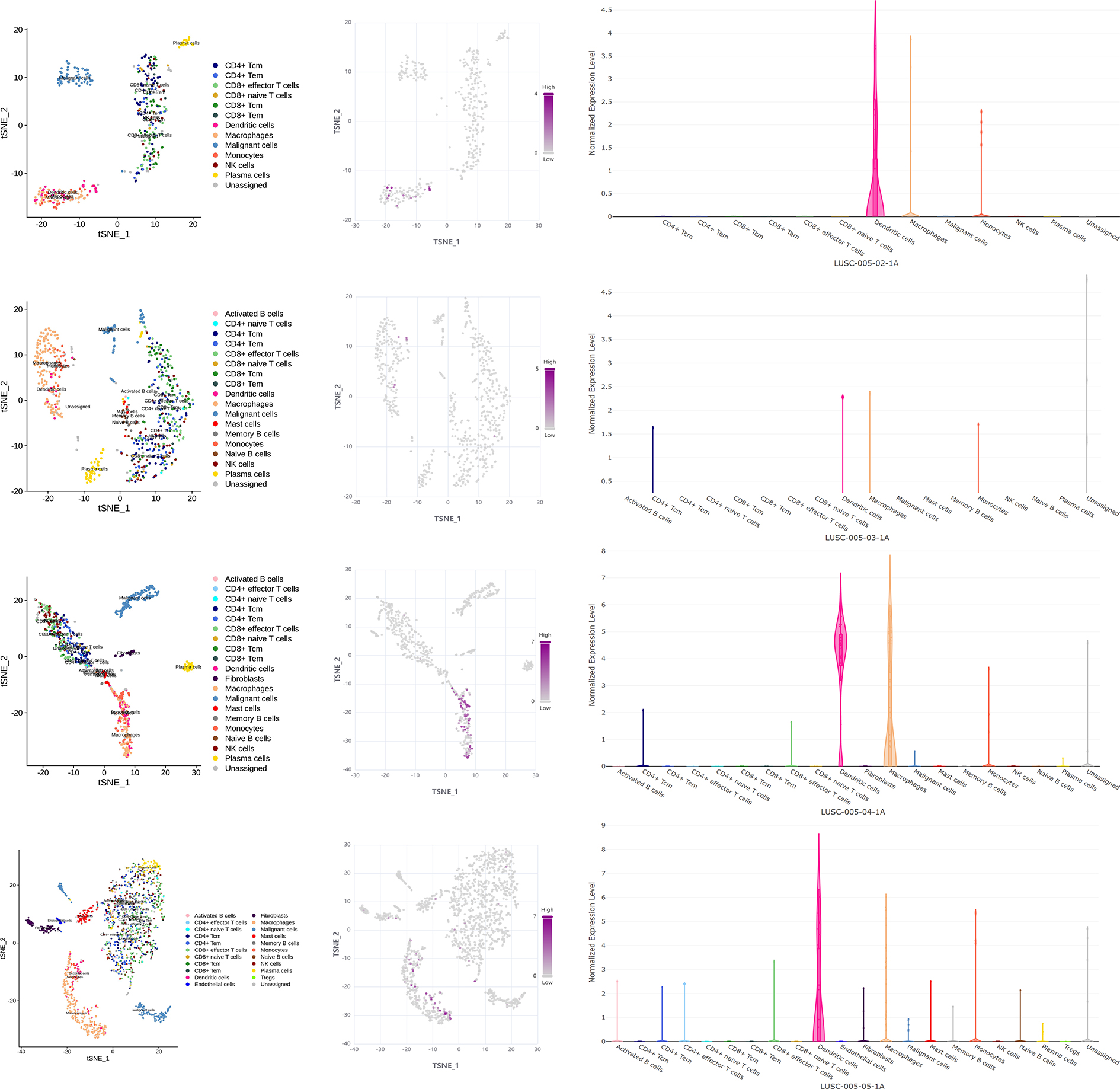

Supplement: Supplemental Information 8 [file peerj-11-15598-s008.jpg]

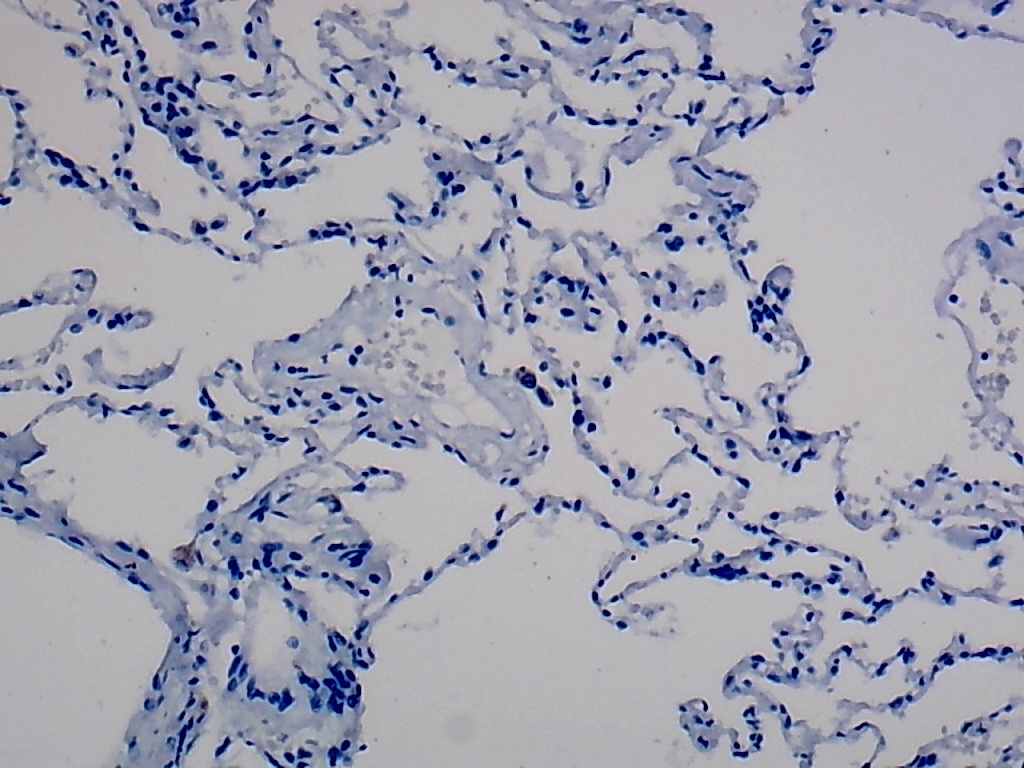

Supplement: Supplemental Information 9 [file peerj-11-15598-s009.jpg]

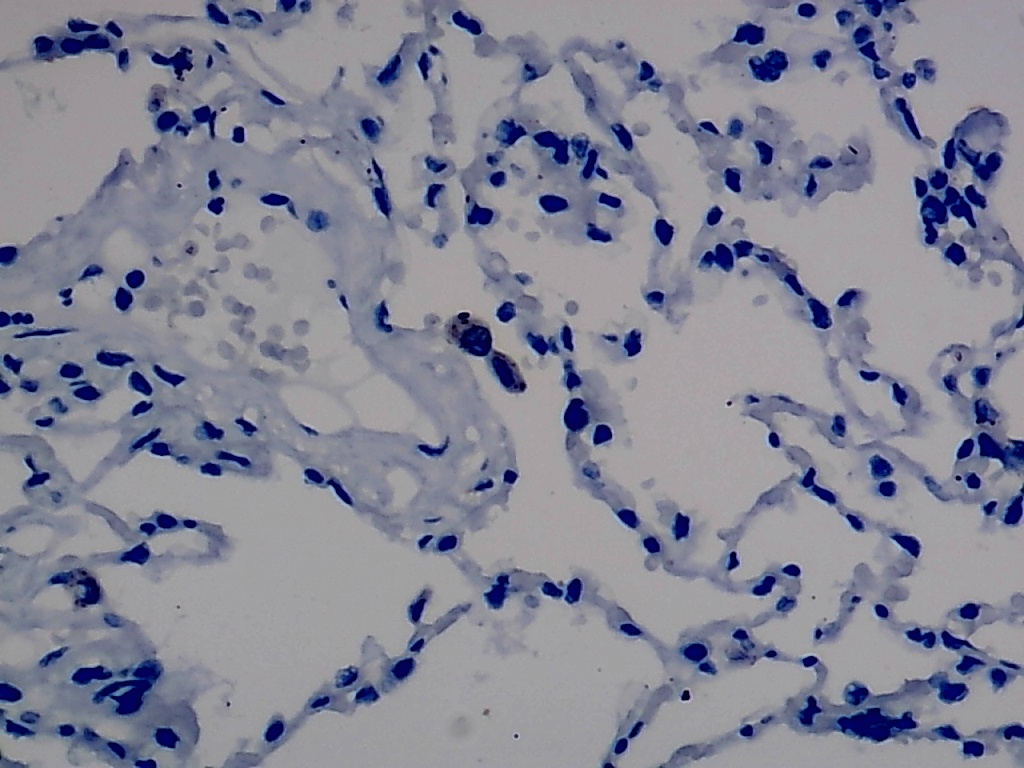

Supplement: Supplemental Information 10 [file peerj-11-15598-s010.jpg]

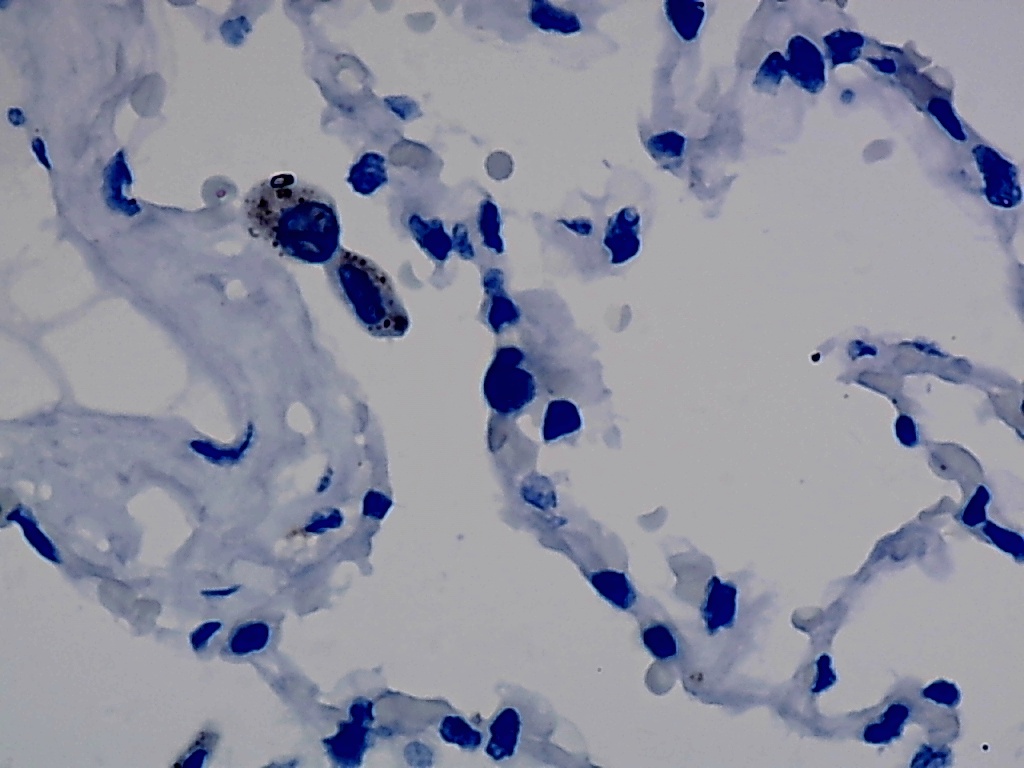

Supplement: Supplemental Information 11 [file peerj-11-15598-s011.jpg]

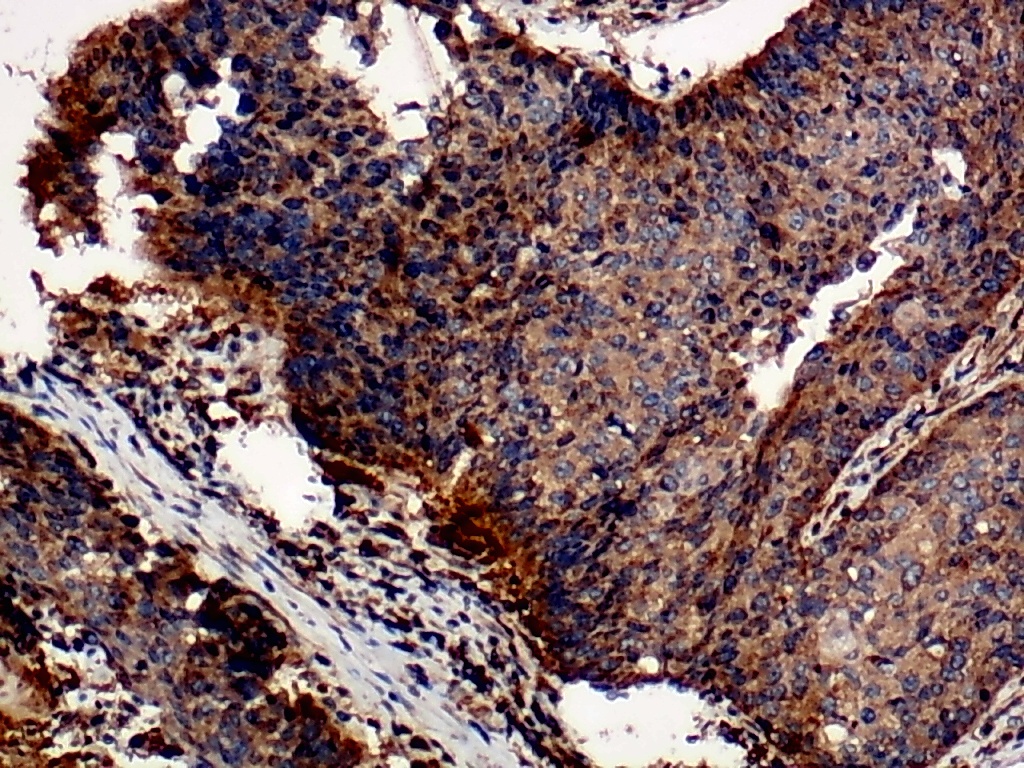

Supplement: Supplemental Information 12 [file peerj-11-15598-s012.jpg]

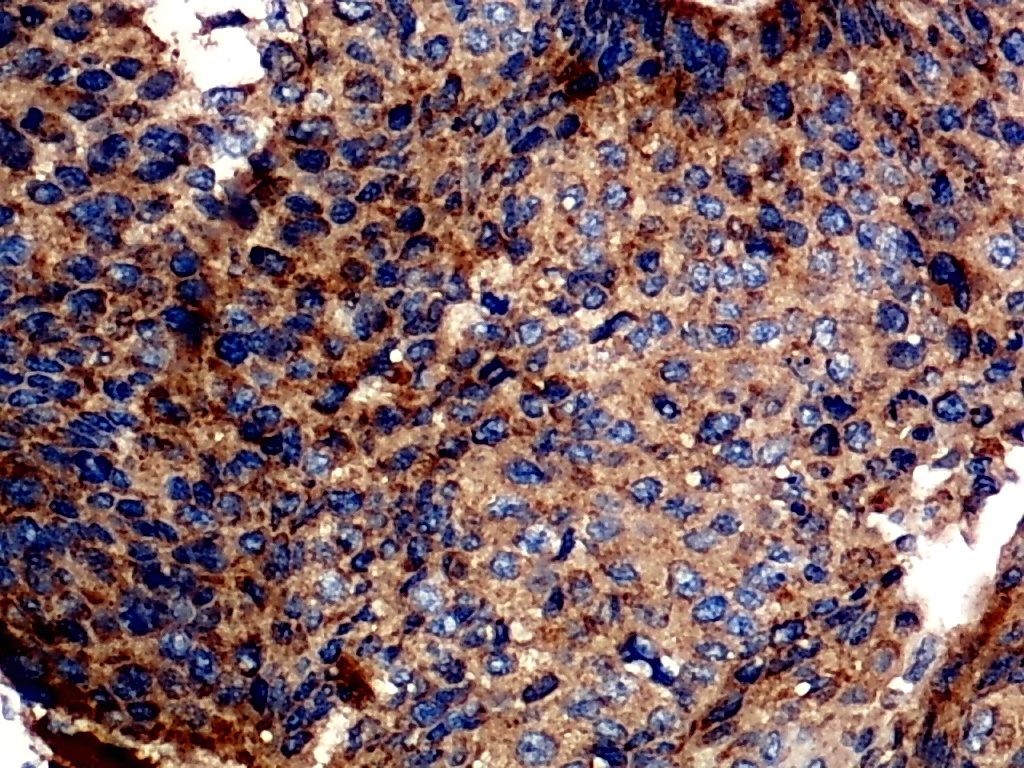

Supplement: Supplemental Information 13 [file peerj-11-15598-s013.jpg]

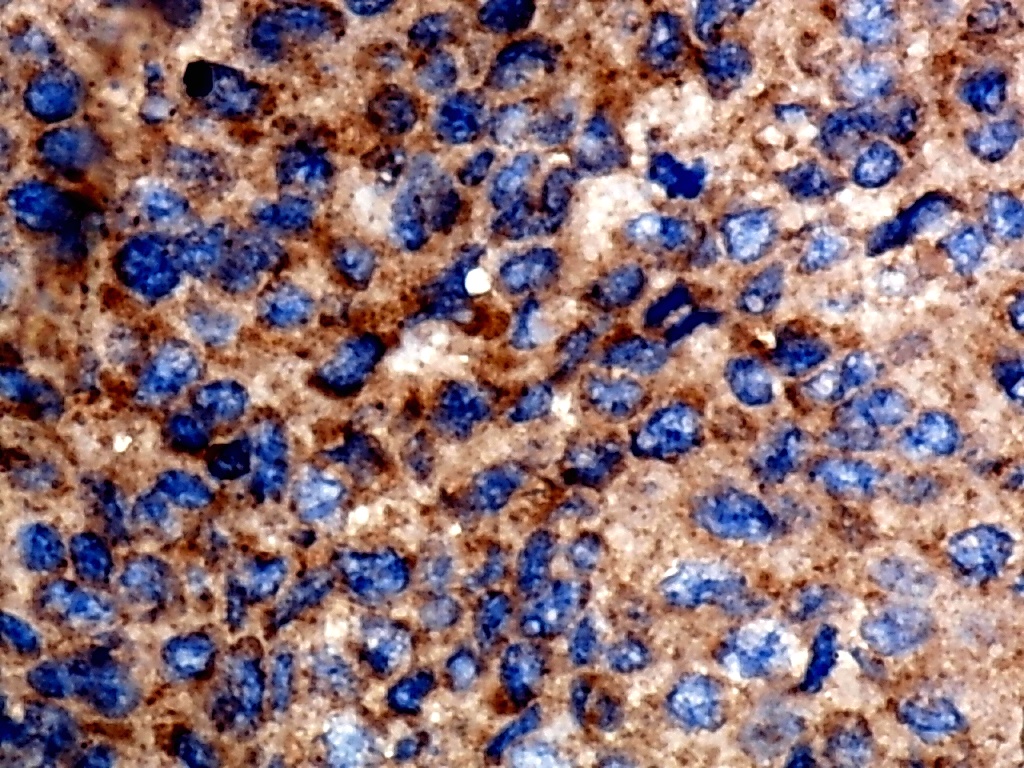

Supplement: Supplemental Information 14 [file peerj-11-15598-s014.jpg]

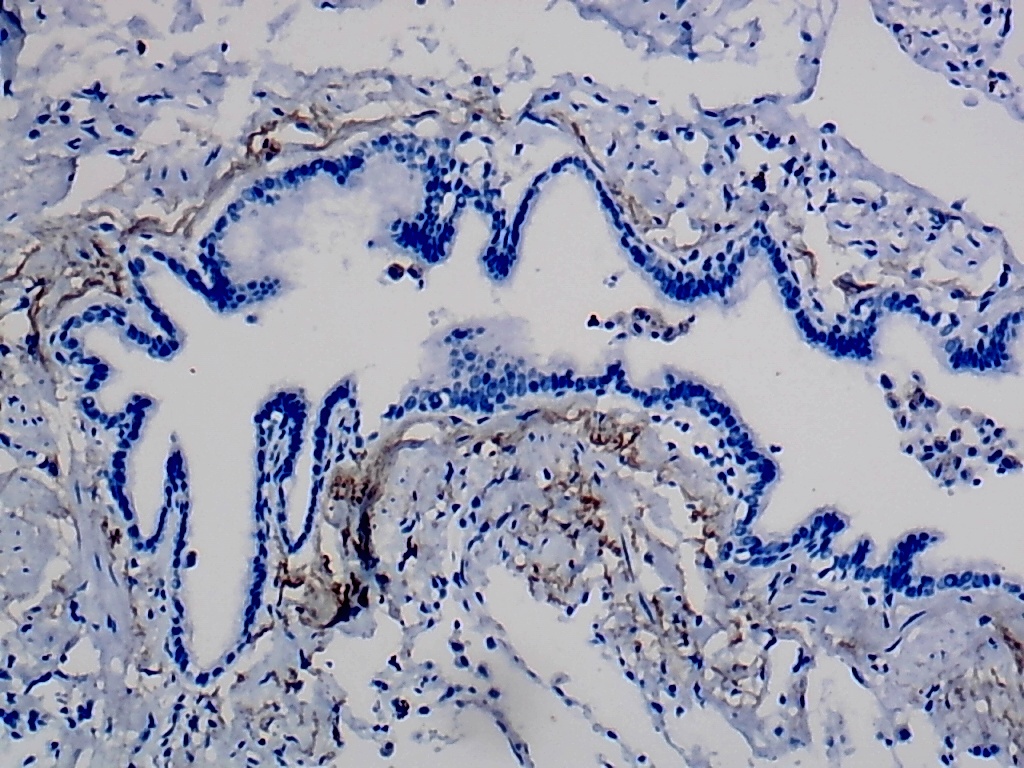

Supplement: Supplemental Information 15 [file peerj-11-15598-s015.jpg]

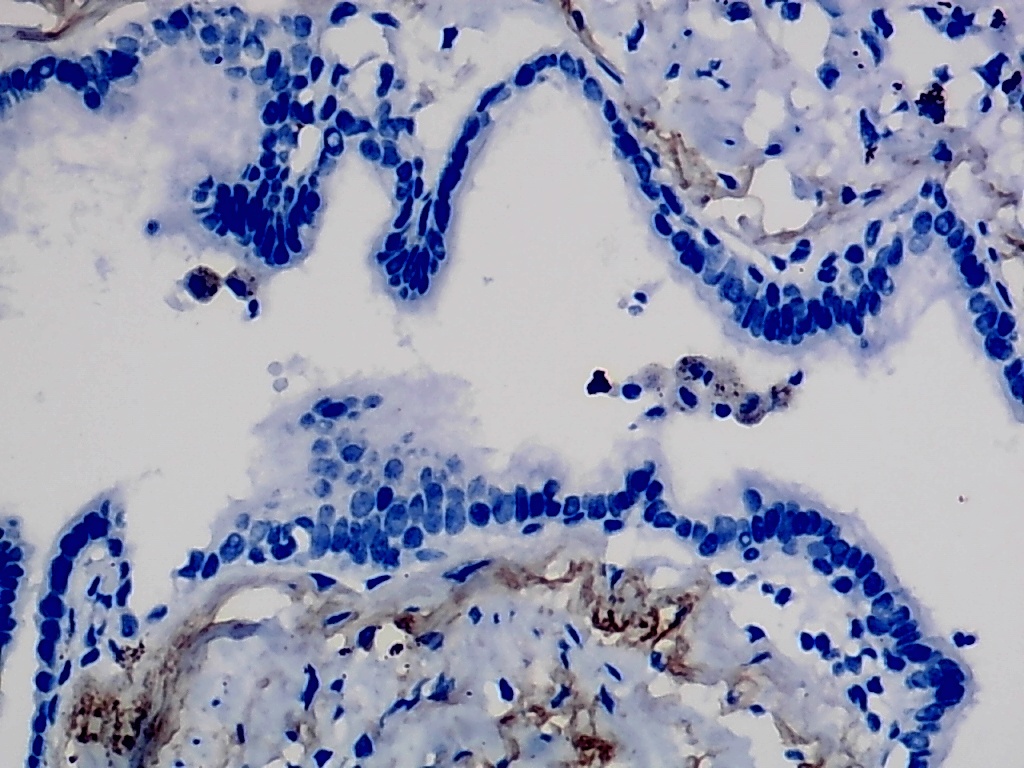

Supplement: Supplemental Information 16 [file peerj-11-15598-s016.jpg]

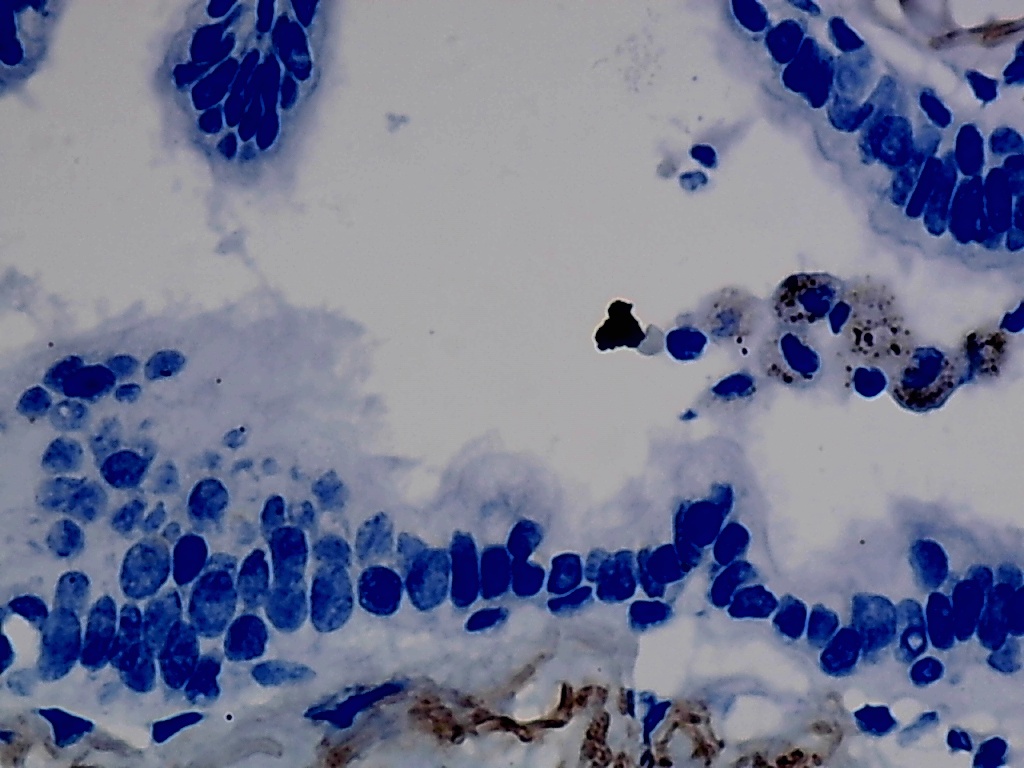

Supplement: Supplemental Information 17 [file peerj-11-15598-s017.jpg]

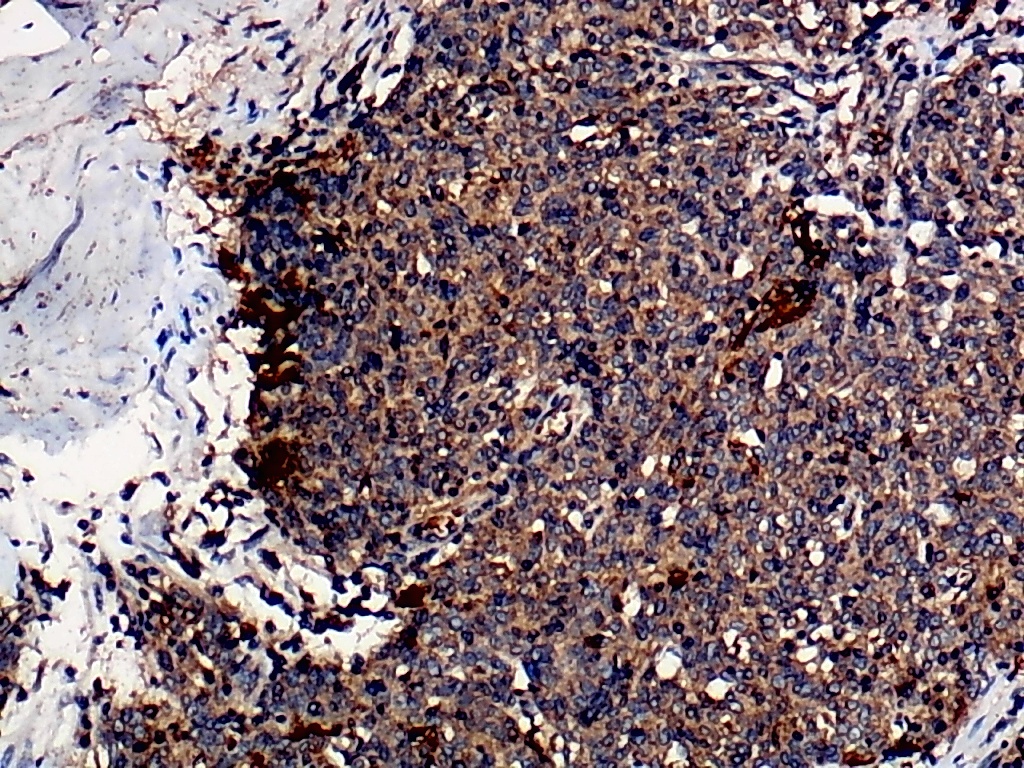

Supplement: Supplemental Information 18 [file peerj-11-15598-s018.jpg]

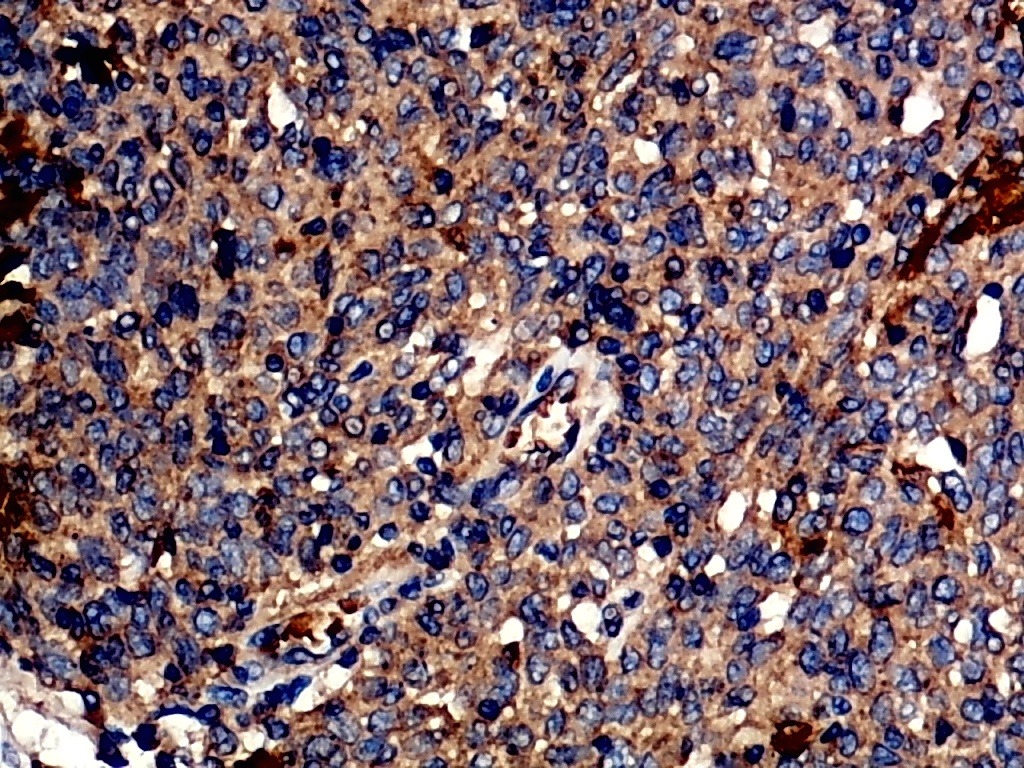

Supplement: Supplemental Information 19 [file peerj-11-15598-s019.jpg]

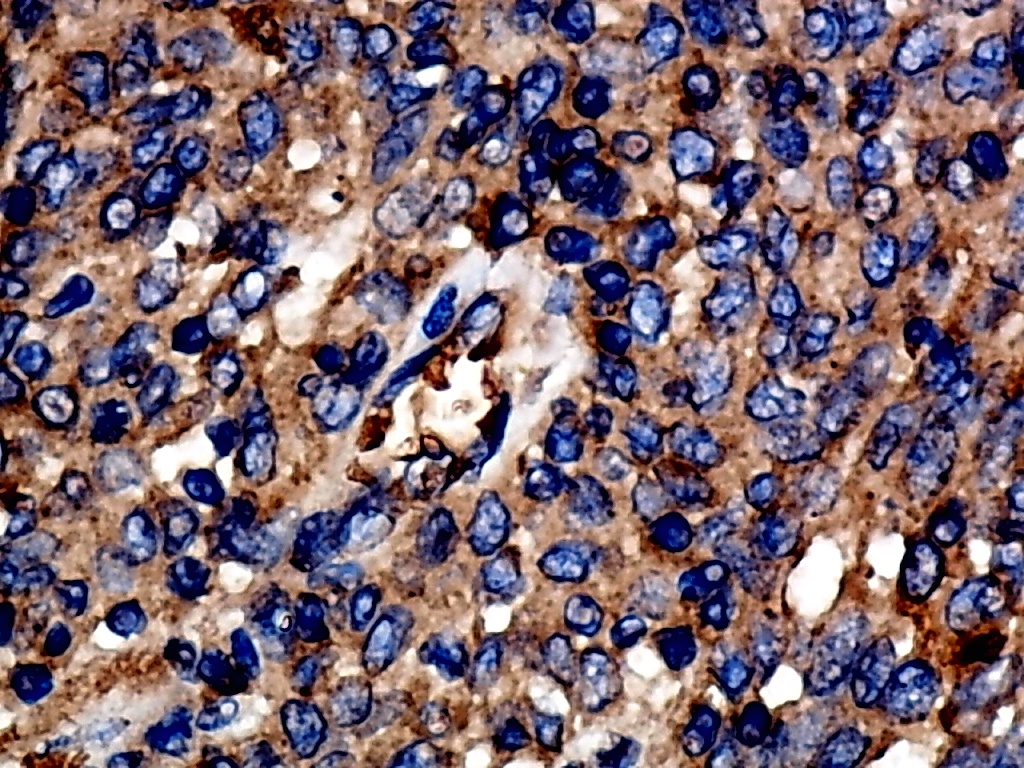

Supplement: Supplemental Information 20 [file peerj-11-15598-s020.jpg]
