# Supplementary material for: MMP12 serves as an immune cell–related marker of disease status and prognosis in lung squamous cell carcinoma
Source: PeerJ. 2023 Aug 16;11:e15598. doi: 10.7717/peerj.15598 (PMC10439720; doi:10.7717/peerj.15598)
Supplement: Supplemental Information 6 [file peerj-11-15598-s006.docx]

**Supplementary material 6.** There are two potential biding sites between SNAI2 motif and the underlying promoter region (upstream 1 kb) of *MMP12*.

| TF | Sequence ID | Start | End | Predicted sequence |
| --- | --- | --- | --- | --- |
| SNAI2 | NC_000011.10:c102875982-102874982 | 470 | 482 | TTCCACGTGTTGT |
| SNAI2 | NC_000011.10:c102875982-102874982 | 766 | 778 | GTCCACCTATCCT |
